# Identification and expression analysis of the small auxin-up RNA (*SAUR*) gene family in *Lycium ruthenicum*

**DOI:** 10.7717/peerj.15941

**Published:** 2023-09-07

**Authors:** Jing Hu, Qiushi Yu, Shengxiu Jiang, Xiaoke Hu, Xuemin Li, Zhongchao Liu

**Affiliations:** 1 State Key Laboratory Breeding Base of Desertification and Aeolian Sand Disaster Combating, Gansu Desert Control Research Institute, Lanzhou, China; 2 Hubei Engineering University, Xiaogan, China

**Keywords:** *Lycium ruthenicum*, MEME analysis, Phylogenetic tree, Gene tissue expression, Plant growth and development

## Abstract

The plant hormone auxin regulates numerous aspects of plant growth and development, and small auxin-up RNA (SAUR) is the largest family of early auxin response genes in higher plants. SAUR has been implicated in the regulation of multiple biological processes. However, no comprehensive analysis of *SAUR* genes has been reported in *Lycium ruthenicum*. *L. ruthenicum* is a thorny shrub with very pronounced salt and drought tolerance, and studies have shown that stem thorns are related to drought tolerance in *L. ruthenicum*. In this study, the identification, phylogenetic analysis, and conserved motif prediction of SAUR genes were extensively explored. Furthermore, the tissue expression patterns of selected SAUR genes were assayed with quantitative real-time polymerase chain reaction (RT-qPCR). A total of 33 putative *LrSAURs* were identified and divided into three clusters in a phylogenetic tree of *L. ruthenicum*. MEME analysis identified 10 motifs in *L. ruthenicum*, and the results suggested that motif 1 and motif 3 were widely distributed. Analyzing the transcriptome data of stem thorns at four developmental stages indicated that *LrSAURs* were differentially expressed in *L. ruthenicum*, and could be divided into six expression patterns. The RT-qPCR analysis of 21 genes showed that *LrSAUR2*, *LrSAUR8*, *LrSAUR9*, *LrSAUR11*, *LrSAUR12*, and *LrSAUR19* were mainly expressed in stems and stem thorns, and may be related to stem thorn development.

## Introduction

Plants in arid desert regions are exposed to severe environmental conditions, and most desert plants are unique and precious. These plants possess constitutive and inducible defense barriers to counteract stresses and have formed various special structures, such as cuticles, waxy layers, trichomes, and spines, during the long process of evolution (Ménard et al., 2013). *Lycium ruthenicum* Murr. (black goji) belongs to the Solanaceae family, and is a unique nutritional and medicinal food mainly distributed in the salinized desert of northwestern China (Liu et al., 2012). Due to its nutritional, medicinal, and ecological value, *L. ruthenicum* has attracted widespread attention. Stem thorns are one of the important symbolic characters of *L. ruthenicum*. Lu, Wang & Fan (2014) found that compared with cultivated plants, wild *L. ruthenicum* had more and denser thorns. Furthermore, Zhang (2019) reported that *L. ruthenicum* did not grow stem thorns when cultured under the conditions of 100% and 80% water-holding capacity (WHC), while plants had a large number of stem thorns under 60% and 40% WHC treatments. These findings indicated that the formation of stem thorns was closely related to soil water content, and drought stress could promote the development of stem thorns in *L. ruthenicum*, suggesting that stem thorns may be a direct response mechanism to drought and one of the important strategies to resist drought stress. However, to date the molecular mechanism of thorn growth induced by drought has not been reported.

Stem thorns develop from axillary buds and can develop into branches under appropriate conditions. Therefore, factors affecting axillary bud development may play an important role in regulating the occurrence of stem thorns. Plant hormones play a key role in axillary meristem formation and the activation and growth of dormant axillary buds (Waldie & Leyser, 2018). Additionally, auxin influences nearly all aspects of plant growth and development by regulating cell division, expansion, differentiation, and patterning *via* regulating the expression of genes (Ren & Gray, 2015). Among auxin response genes, the small auxin-up RNA (SAUR) is the largest family of auxin early response genes, which are rapidly induced by auxin and encode plant-specific small proteins (Ren & Gray, 2015). SAURs are crucial regulators of diverse aspects of plant growth, development, and stress responses, such as root development, hypocotyl elongation, leaf growth and senescence, and response to drought, low temperature, disease and insect pests (Kant et al., 2009; Abbas, Alabadi & Blazquez, 2013; Vanhaeren et al., 2014; Ren & Gray, 2015). In *Arabidopsis thaliana*, the expression levels of *AtSAUR36* and *AtSAUR41* were closely related to cell expansion, and overexpression of these genes significantly elongated hypocotyl epidermal cells (Chae et al., 2012; Spartz et al., 2012; Spartz et al., 2017; Kong et al., 2013; Stamm & Kumar, 2013). SAUR32, SAUR19 and SAUR36 are mainly related to the formation of the apical hook, and overexpression seedlings have short hypocotyls in *A. thaliana* (Park et al., 2007; Spartz et al., 2012; Stamm & Kumar, 2013). AtSAUR41 and AtSAUR76 are related to root development, and the up-regulation of their transcription can promote taproot elongation and lateral root development (Kong et al., 2013; Markakis et al., 2013). The up-regulated expression of *SAUR12*, *SAUR34*, *SAUR54*, *SAUR67*, *SAUR91*, and *SAUR97* in poplar improved the adaptability of seedlings to low temperature (Hu et al., 2018). In addition, SAUR family proteins are regulated by miR159, which promotes drought tolerance in wheat (Gupta et al., 2014). Moreover, SAUR30 is also related to drought adaptation in poplar (Chen, Hao & Cao, 2014). Research suggests that SAURs are crucial regulators of diverse aspects of plant growth and development. Furthermore, in *A. thaliana*, *SAUR19* overexpression and *SAUR19/23/24* amiRNA knockdown seedlings showed increased and decreased basipetal indole-3-acetic acid (IAA) transport in hypocotyls, respectively (Spartz et al., 2012). In contrast, the overexpression of *OsSAUR39* in rice resulted in reduced IAA transport (Kant et al., 2009), indicating that SAUR proteins are capable of modulating IAA transport. Although SAUR family identification and expression analysis have been conducted in a variety of plants, the functions of the members of this gene family are largely unknown, and whether IAA and SAUR are involved in regulating the growth of stem thorns under drought conditions is not yet known. Hence, further study of SAURs to reveal their biological effects in *L. ruthenicum* is valuable.

In this study, the salt-xerophyte *L. ruthenicum*, which produces a large number of stem thorns under drought conditions, was used as a material. *SAUR* family members were identified and the tissue expression patterns of genes in *L. ruthenicum* were analyzed. The findings of this study provide valuable information on the stress-response profiles of *SAUR* genes in *L. ruthenicum* and lay a solid foundation for elucidating the functions of *SAUR* genes in regulating the generation of stem thorns.

## Materials and Methods

### Plant growth conditions and treatments

*L. ruthenicum* seeds were collected from Minqin County (101°59′E–104°12′E, 38°08′N–39°26′N), in Gansu Province of northwest China. Seedlings were cultured as described by Hu et al. (2022) with minor modifications: when seedlings grew to about 1 cm, they were transplanted into plant pots (20 cm^3^; three seedlings/container) filled with nutrient soil. Seedlings with consistent growth were selected after 20 days of cultivation and then sprayed with 0, 25, and 50 mg/L IAA every day for 60 days. To minimize the effects of possible environmental gradients in the greenhouse, pots were randomly reassigned to new positions every day.

### Transcriptome analysis

Seedlings of *L. ruthenicum* were cultured for three weeks and then divided into two treatments: 80% WHC and 40% WHC. After treatment about for 60 days, seedlings were divided into treatment groups as follows: (i) D1: Non-thorn stem nodes near the apex under the treatment of 40% WHC. (ii) D2: Sprout-thorn stem nodes under the treatment of 40% WHC. (iii) D3: Long green thorn stem nodes under the treatment of 40% WHC. (iv) C: Stem nodes under the treatment of 80% WHC taken from the same part as that of group (iii).

Four groups of samples were mixed or used separately for third or second-generation transcriptome sequencing (Biomarker Technologies Company, China). This experiment was performed with three biological replicates. Raw data were submitted to the public NCBI Sequence Read Archive database (https://www.ncbi.nlm.nih.gov/sra, accession number SRR22514555).

### Sequence database search and identification of the *LrSAURs* in *L. ruthenicum*

The potential annotated nucleotide sequences of *LrSAURs* were downloaded from the NCBI Sequence Read Archive database (https://www.ncbi.nlm.nih.gov/sra, accession number SRR22514555). First, SAUR protein sequences from *A. thaliana* were collected (https://www.uniprot.org/) to construct a local BLASTP protein database (with an *E*-value cut off of 10^−10^ and an identity of 50%). Next, a hidden Markov Model (HMM) was constructed with the HMMER 3.0 program to find all predicted *SAUR* family members of *L. ruthenicum.* Then, the aligned sequences were considered as candidate *SAUR* family sequences, and the candidate *LrSAURs* were named beginning with “Lr” for *L. ruthenicum*. ProtParam (http://web.expasy.org/protparam/) was used to analyze the physicochemical parameters (length, molecular weight, and isoelectric point) of the candidate *LrSAURs*. Eighty-three *A. thaliana* protein sequences were downloaded from https://www.arabidopsis.org/, and fifty-eight rice (*Oryza sativa*) SAUR genes (OsSAURs) were searched according to the Data S1 in a previous report (Jain, Tyagi & Khurana, 2006). Multiple alignment using fast Fourier transform (MAFFT: http://mafft.cbrc.jp/alignment/software/) was used to analyze the multiple sequence alignment among these *LrSAURs*. A tree was constructed using the neighbor-joining (NJ) method in MEGA 7.0 with partial deletion and the p-distance model.

### Structural characterization and heatmap analysis of *LrSAURs*

The MEME program was used to identify the conserved protein motifs of *LrSAURs*, with optimum motif widths of 6–50 residues and a maximum of 10 motifs. For the analysis of gene expression, the number of clean tags for each gene was calculated and normalized to fragments per kilobase of transcript per million mapped reads (FPKM): FPKM = {cDNA Fragments \over {Mapped Fragments (Millions) * Transcript Length (kb)}}. Heatmaps of genes in the different stem thorn development stages were generated based on their FPKM values using the TBtools (v0.67373) software (https://github.com/CJ-Chen/TBtools).

### RNA extraction, cDNA synthesis, and quantitative real-time PCR (RT-PCR) analysis

Total RNA was extracted with a Trizol Kit R6827-01 (Omega Bio-Tek, USA) according to the manufacturer’s instructions from the roots, stems, stem thorns and leaves of three-month-old *L. ruthenicum* seedlings. A NanoDrop 2000 instrument (Thermo Fisher Scientific, Waltham, MA, USA) was used to evaluate the RNA quantity and quality (A260 nm/A280 nm: 1.8–2.0 for purity; 500–1000 ng/µL for concentration). The Evo M-MLV RT Kit AG11728 (Accurate Biotechnology, Changsa, China) was used to reverse-transcribe the total RNA into cDNA according to the instructions. Twenty-one *LrSAURs* were selected from the 33 genes according to the FPKM values (FPKM ≥ 5 at least one of the thorn developmental stages) for the RT-qPCR analysis of the tissue expression. The reference gene used was *LrEF1 α* (JX427553) (Tiika et al., 2020). The primer pairs are shown in Table S1. RT-qPCR analysis was performed using the SYBR Green Premix Pro Taq HS (Accurate Biotechnology, Changsha, China) according to the manufacturer’s protocol. Analysis was run on the QuantStudio 5 Real-Time PCR Instrument (ABI) (Life Technologies Holdings). The cycling parameters were 95 °C for 5 min, followed by 40 cycles of 95 °C for 10 s, and 60 °C for 30 s. The data was quantitated using the 2^−ΔΔ*Ct*^ method (Livak & Schmittgen, 2001). This experiment was performed with three biological replicates.

### Data analysis

Values of the gene expression levels were presented as the means ± SE (*n* = 3). Data were analyzed using one-way analysis of variance followed by Duncan’s multiple range tests (*p* ≤ 0.05) (SPSS statistical software, Version 25.0; SPSS Inc., Chicago, IL, USA).

## Results

### Effects of IAA on the stem thorn development of *L. ruthenicum*

**Figure 1:**
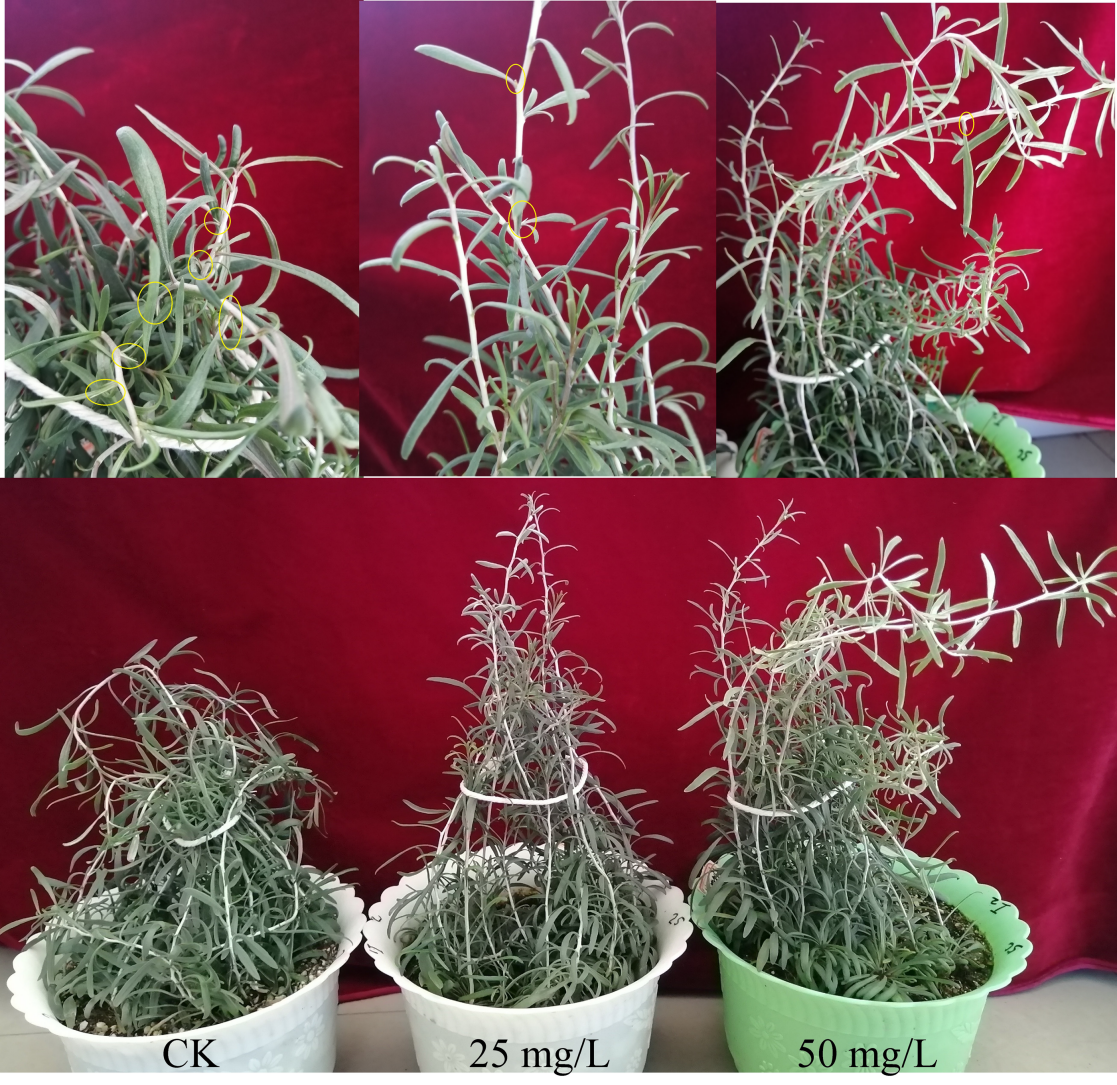
Effects of indole-3-acetic acid (IAA) (0, 25, and 50 mg/L) on stem thorn development in *Lycium ruthenicum*. The thorns are encircled with yellow lines.

The plant heights under the 25 and 50 mg/L IAA treatments were taller than those under the control (CK). A large number of stem thorns appeared in CK plants, a small number of thorns appeared later in 25 mg/L IAA plants, and fewer thorns were found in the 50 mg/L IAA plants than in the 25 mg/L IAA treatment (Fig. 1).

### Identification of *LrSAUR* s

A total of 33 putative *LrSAURs* with a central domain (PF02519) were acquired and named *LrSAUR1*–*LrSAUR33* (Table 1). The obtained gene lengths ranged from 548 bp (*LrSAUR13*) to 7237 bp (*LrSAUR19*), and the predicted amino acids ranged from 82 aa (*LrSAUR23*) to 586 aa (*LrSAUR1*) (Table 1). In addition, the theoretical isoelectric point (pI) of the *LrSAURs* ranged from 5.27 to 10.18, and the molecular weight (Mw) ranged from 9202.04 to 67625.88 Da.

**Figure 2:**
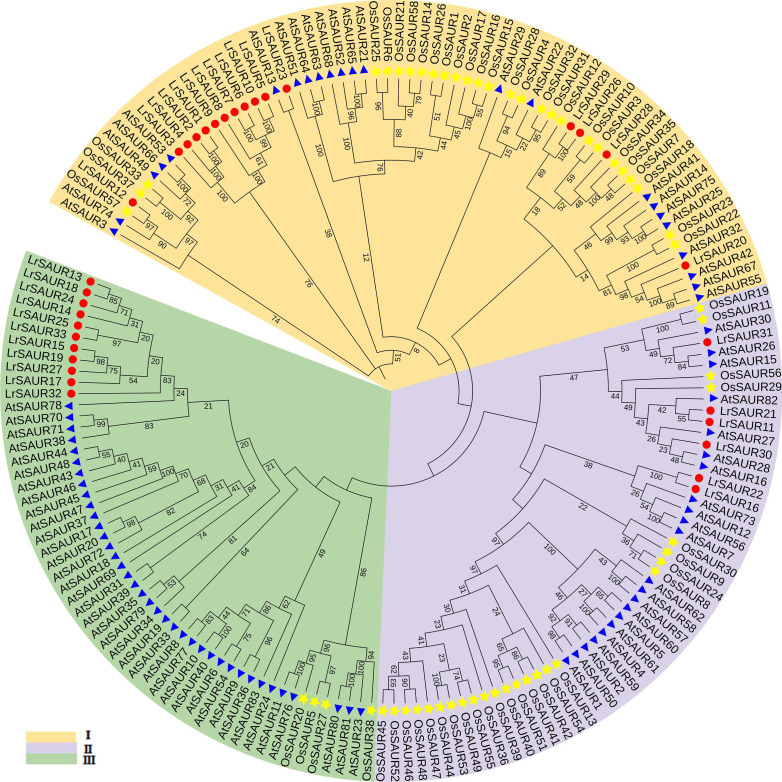
Phylogenetic relationship of LrSAURs, AtSAURs, OsSAURs. LrSAURs from *Lycium ruthenicum*, AtSAURs from *Arabidopsis thaliana*, and OsSAURs from rice. The different colored arcs indicate different groups, and red circles, blue triangles and yellow stars represent LrSAURs, AtSAURs and OsSAURs, respectively.

Phylogenetic analysis of SAUR genes is an effective way to unveil the functions of uncharacterized SAURs, and 174 SAUR protein sequences from *L. ruthenicum*, Arabidopsis and rice were aligned to perform a phylogenetic tree (Fig. 2). According to the phylogenetic tree, these proteins can be divided into three groups based on their sequence similarities with orthologs in other plants (Fig. 2). Group I, II and III contained 16, six and 11 LrSAUR members, respectively. The OsSAUR proteins were mainly placed in Group I and II, most of AtSAURs were in Group I and II.

**Table 1:** Statistical information of LrSAURs in *L. ruthenicum*.

Gene name	Amino acids	MW (Da)	pI	Instability index	Aliphatic index	GRAVY
LrSAUR1	586	67625.88	7.15	47.64	84.06	−0.621
LrSAUR2	338	38866.58	7.00	46.50	80.24	−0.653
LrSAUR3	397	44228.90	5.52	46.89	75.29	−0.527
LrSAUR4	199	22906.93	5.27	41.02	75.53	−0.734
LrSAUR5	219	24647.28	5.71	51.05	45.43	−1.533
LrSAUR6	228	25699.56	5.82	45.33	45.79	−1.560
LrSAUR7	224	25184.98	5.81	45.96	46.61	−1.521
LrSAUR8	237	26828.93	6.19	48.95	47.30	−1.530
LrSAUR9	198	22342.65	5.38	54.71	48.79	−1.530
LrSAUR10	214	24108.67	5.49	48.55	44.67	−1.556
LrSAUR11	122	14354.17	6.47	41.11	71.89	−0.694
LrSAUR12	127	14870.92	7.79	58.51	91.26	−0.670
LrSAUR13	86	9666.26	8.93	35.45	88.37	−0.079
LrSAUR14	85	9607.08	6.71	43.13	88.24	−0.074
LrSAUR15	86	9781.42	5.73	42.32	96.28	0.016
LrSAUR16	138	16012.81	8.89	48.48	88.91	−0.319
LrSAUR17	102	11522.63	9.52	36.56	101.18	0.160
LrSAUR18	86	9637.22	7.92	39.81	87.33	−0.077
LrSAUR19	86	9781.42	5.73	42.32	96.28	0.016
LrSAUR20	148	16725.27	9.79	65.03	82.30	−0.295
LrSAUR21	133	15525.56	6.55	28.80	82.78	−0.616
LrSAUR22	138	16012.81	8.89	48.48	88.91	−0.319
LrSAUR23	82	9202.04	10.18	29.87	112.80	0.323
LrSAUR24	85	9657.30	8.89	41.11	88.24	−0.076
LrSAUR25	83	9489.96	8.89	38.91	77.47	−0.349
LrSAUR26	100	11767.53	7.92	46.95	93.50	−0.543
LrSAUR27	95	10704.51	7.95	37.44	92.21	0.083
LrSAUR28	160	18312.90	9.10	29.72	61.50	−0.559
LrSAUR29	100	11709.49	8.61	49.42	93.50	−0.505
LrSAUR30	111	12919.77	8.62	50.90	79.82	−0.638
LrSAUR31	164	19089.01	10.06	40.92	74.21	−0.514
LrSAUR32	83	9457.93	6.06	44.07	92.77	−0.095
LrSAUR33	83	9549.19	8.89	37.88	86.75	−0.081

**Figure 3:**
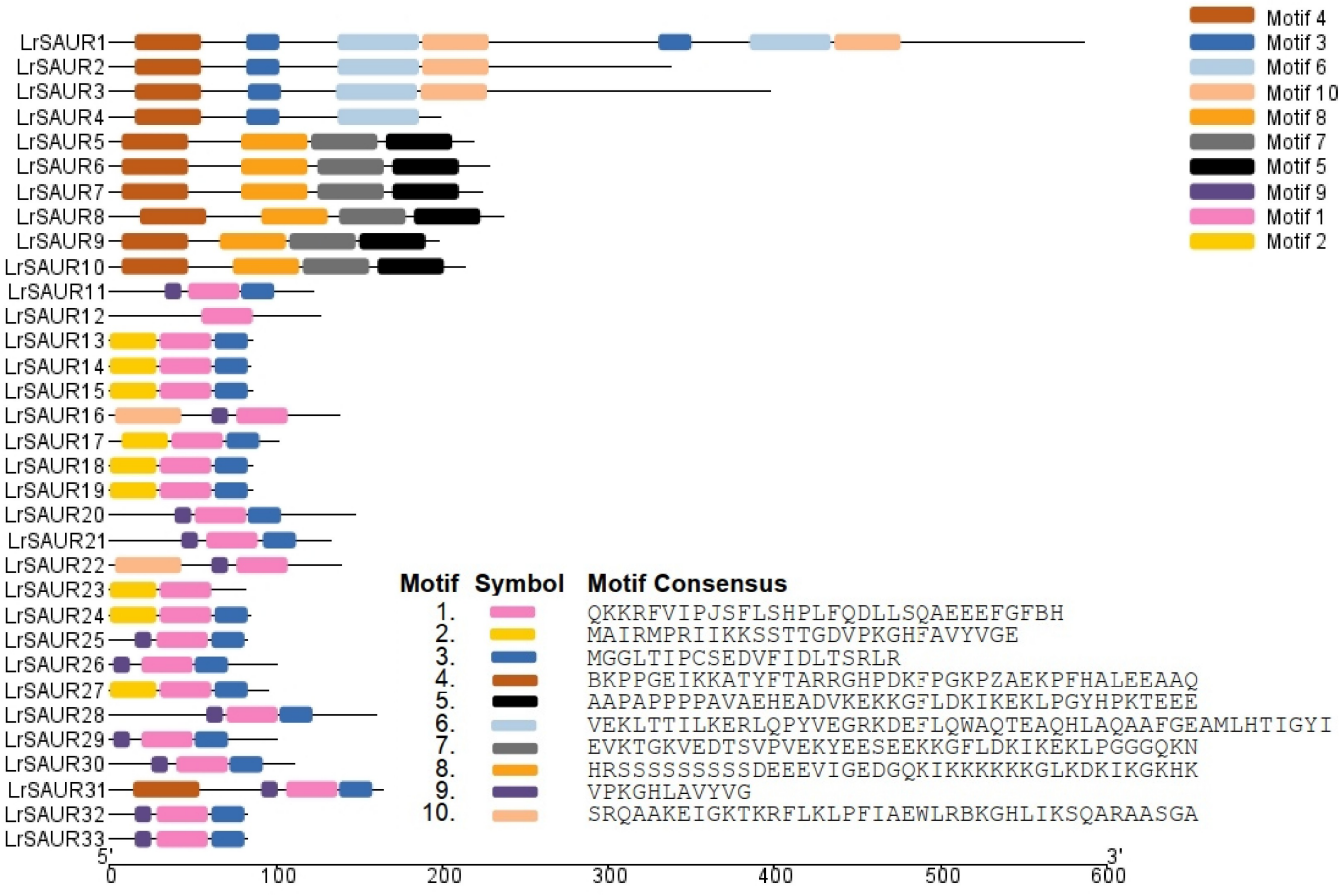
Prediction of conserved motifs of LrSAURs in *Lycium ruthenicum*. Thirty-three LrSAURs are used to predict the motifs by MEME. Different motifs and their position in each LrSAUR sequence are represented by different colored boxes.

MEME software was used to analyze the LrSAUR sequences, and a total of 10 conserved motifs were identified: motif 1–motif 10 (Fig. 3). As shown in Fig. 3, the number and type of conserved motifs contained in 33 LrSAUR proteins were different: one LrSAUR protein contained one conserved motif, one LrSAUR protein contained two conserved motifs, 21 LrSAUR proteins contained three conserved motifs, nine LrSAUR proteins contained four conserved motifs, and one LrSAUR protein contained seven conserved motifs. Motif 1 and motif 3 were widely distributed.

### Expression analysis of *LrSAURs* under drought treatment

**Figure 4:**
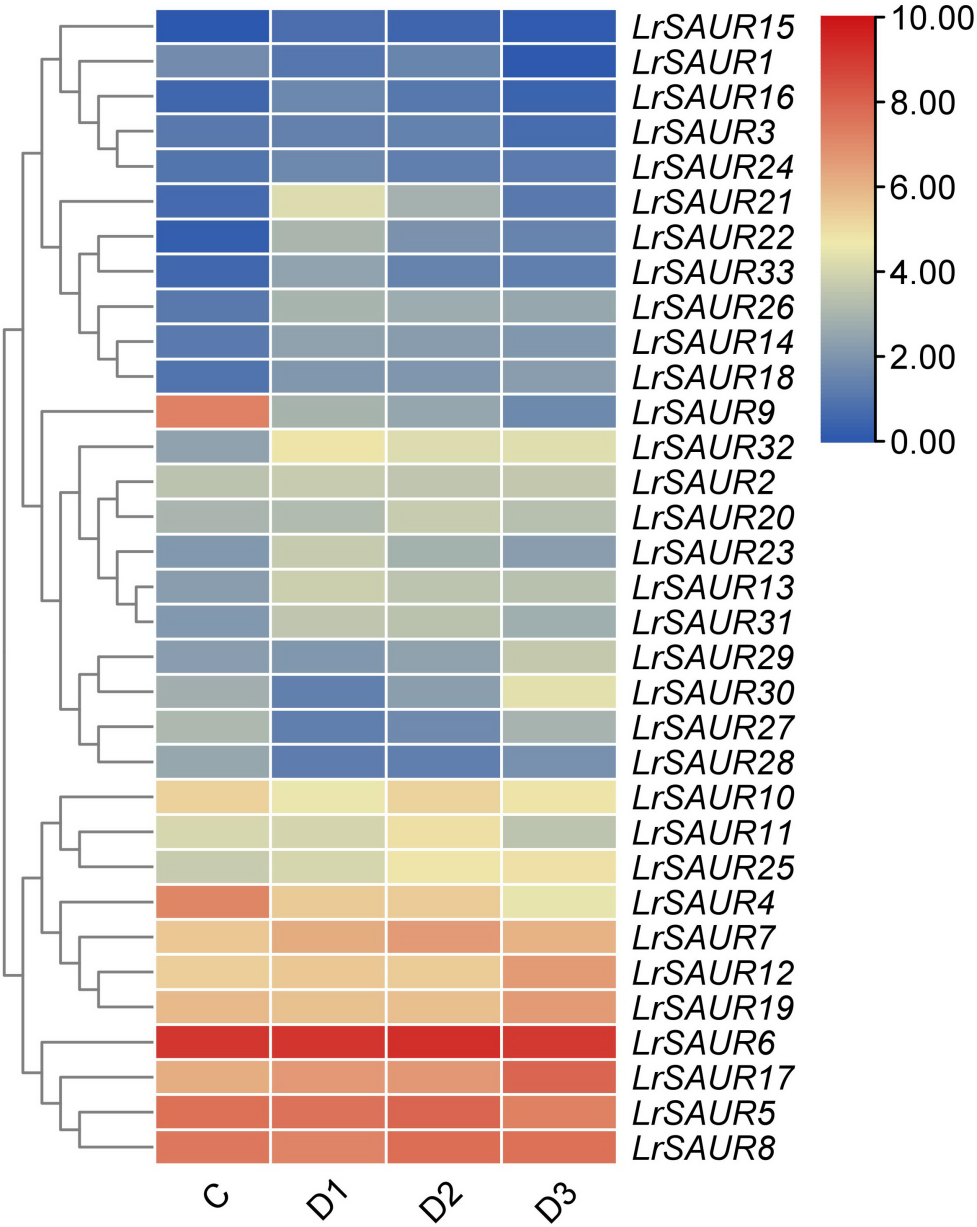
Heatmap of *LrSAUR* s in different developing thorns. The expression profiles of *LrSAUR* s are based on FPKM-values from the RNA sequencing data. Low and high expression levels of genes are indicated by blue and red boxes.

The heatmap illustration of expression profiles of *LrSAURs* in different thorn developmental stages is shown in Fig. 4. Thirty-three *LrSAURs* were found to exhibit different expression patterns in *L. ruthenicum*. The clustering results showed that these genes could be divided into six patterns. *LrSAUR1*, *LrSAUR3*, *LrSAUR14*, *LrSAUR15*, *LrSAUR16*, *LrSAUR18*, *LrSAUR22*, *LrSAUR24*, *LrSAUR26*, and *LrSAUR28* were at low expression levels at all developmental stages. The expression level of *LrSAUR21* was up-regulated once the first thorn had grown and down-regulated when thorns grew long. The transcript abundance of *LrSAUR9* was higher in the control, but it was down-regulated gradually with the development of stem thorns. *LrSAUR32*, *LrSAUR2*, *LrSAUR20*, *LrSAUR23*, *LrSAUR13*, and *LrSAUR31* gradually increased with the emergence of thorns and then showed a downward trend. The expression abundance of *LrSAUR29*, *LrSAUR30*, *LrSAUR27*, and *LrSAUR28* decreased first and then increased with the growth of thorns. Moreover, *LrSAUR4*, *LrSAUR5*, *LrSAUR6*, *LrSAUR7*, *LrSAUR8*, *LrSAUR12*, *LrSAUR17*, *LrSAUR19*, *LrSAUR25*, and *LrSAUR32* had high expression levels in different stages of stem thorn development of *L. ruthenicum*.

**Figure 5:**
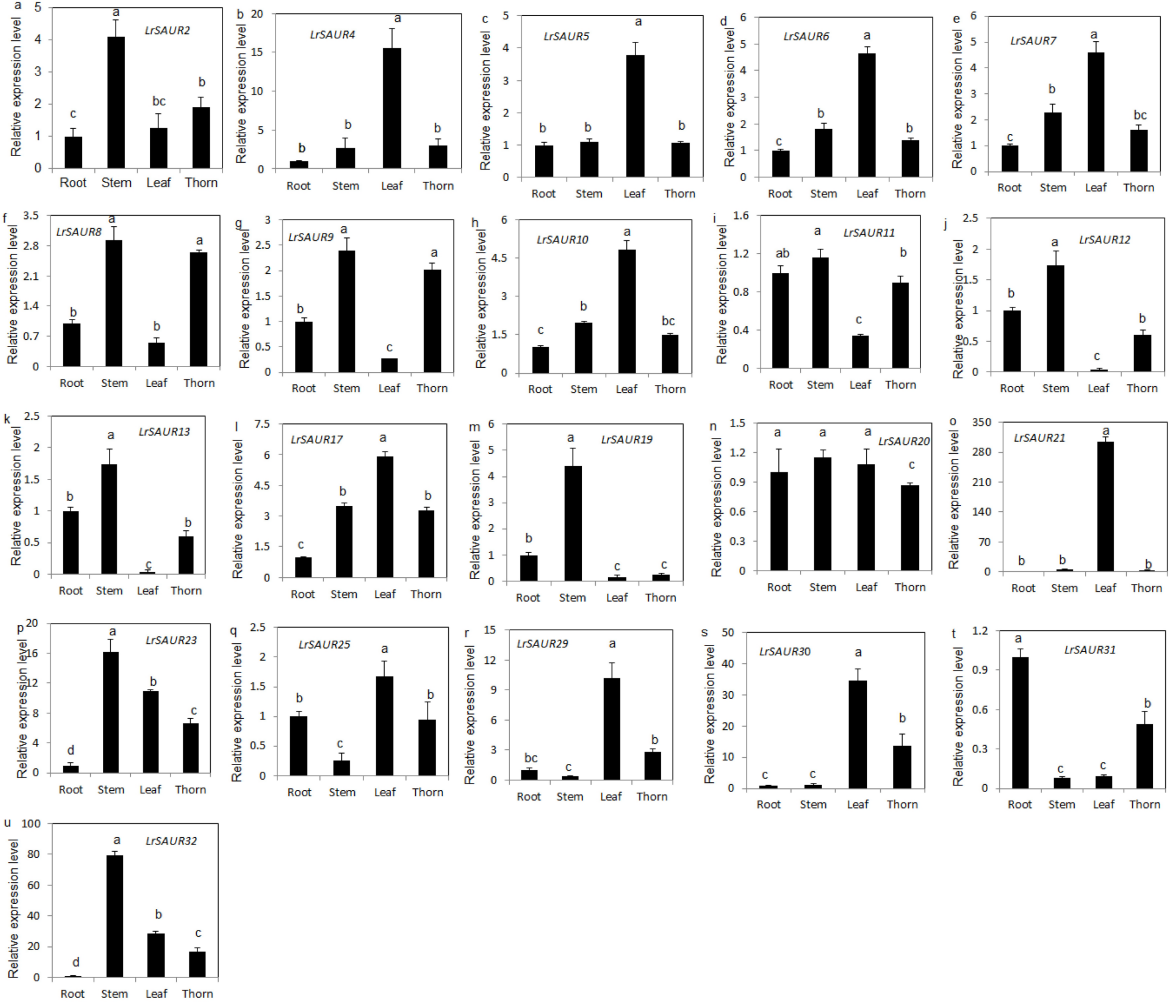
Tissue expression profiles of *LrSAURs* in *Lycium ruthenicum* based on Real-time qPCR. Different lowercase letters indicate significant differences (*p* < 0.05).

Twenty-one genes were selected from the *LrSAURs* for tissue expression analysis using RT-qPCR (FPKM ≥ 5) (Fig. 5). All the *LrSAURs* had different expression patterns: *LrSAUR31* was mainly expressed in roots, while *LrSAUR13*, *LrSAUR23*, and *LrSAUR32* were primarily expressed in stems and leaves. *LrSAUR4*, *LrSAUR5*, *LrSAUR6*, *LrSAUR7*, *LrSAUR10*, *LrSAUR21*, *LrSAUR25*, *LrSAUR29*, and *LrSAUR30* were predominantly expressed in leaves. *LrSAUR2*, *LrSAUR8*, *LrSAUR9*, *LrSAUR11*, *LrSAUR12*, and *LrSAUR19* were mainly expressed in stems and stem thorns.

**Figure 6:**
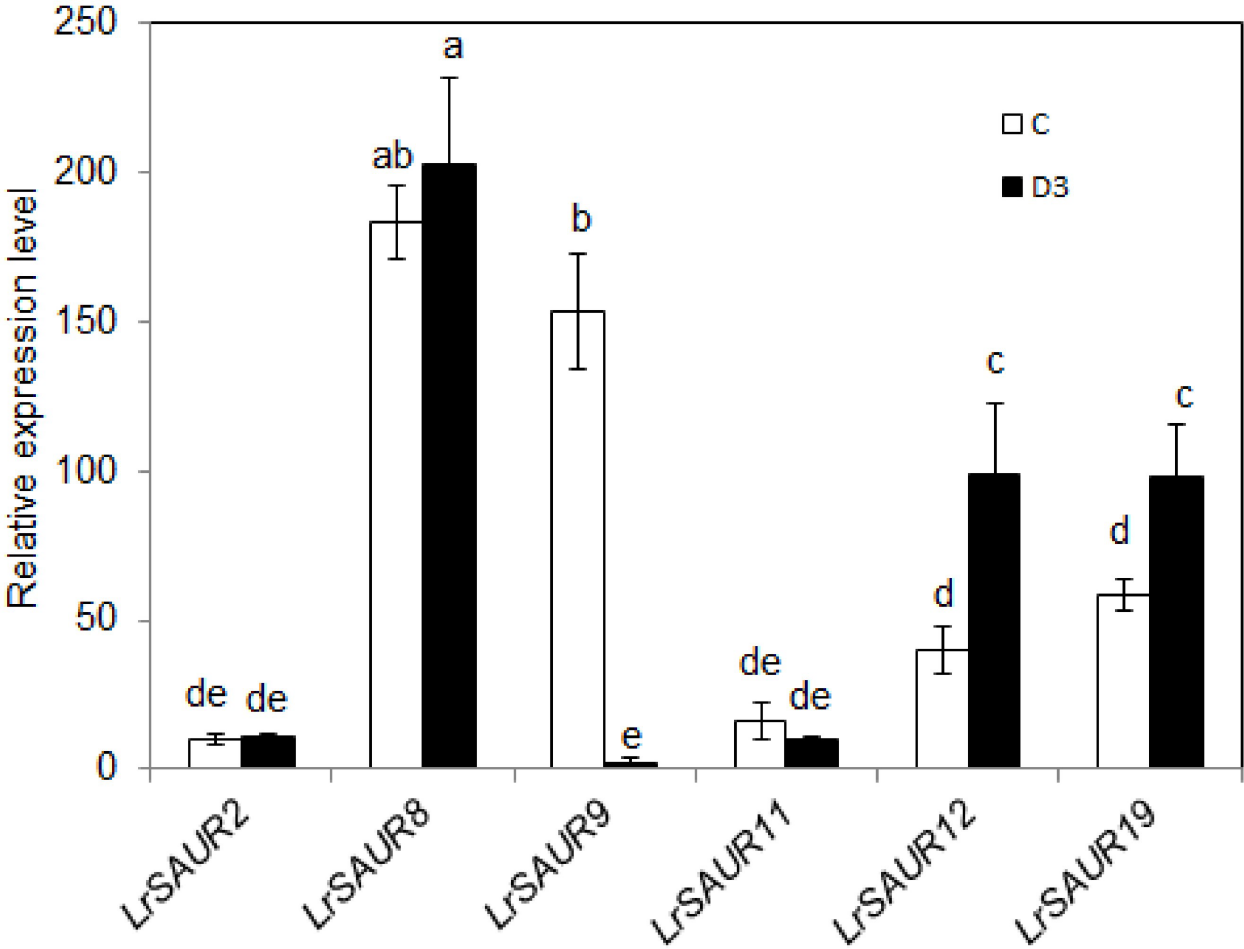
Expression profiles of *LrSAUR2*, *LrSAUR8*, *LrSAUR9*, *LrSAUR11*, *LrSAUR12*, *LrSAUR19* in the C and D3 treatments. The expression profiles of *LrSAUR* s are based on FPKM-values from the RNA sequencing data. Different lowercase letters indicate significant differences (*p* < 0.05).

To further verify the genes mainly expressed in stems and thorns, this work analyzed the expression levels of *LrSAUR2*, *LrSAUR8*, *LrSAUR9*, *LrSAUR11*, *LrSAUR12* and *LrSAUR19* from the C and D3 transcriptome samples (Fig. 6). Compared with the C treatment, *LrSAUR9* was significantly down-regulated under treatment D3, while *LrSAUR12* and *LrSAUR19* were significantly up-regulated, and *LrSAUR2*, *LrSAUR8*, and *LrSAUR11* showed no significant changes.

## Discussion

Plant hormones play important roles in axillary meristem formation and activation and axillary bud outgrowth. Studies have indicated that auxin, cytokinin, and strigolactone are involved in regulating shoot branching (Waldie, McCulloch & Leyser, 2014; Wang et al., 2018). The polar transport of IAA from the stem apex to the base inhibits the growth of axillary buds, resulting in apical dominance. If the terminal bud is removed, axillary bud growth is activated; when IAA is applied to the site of terminal bud removal, axillary bud growth will be re-inhibited (Müller & Leyser, 2011). In the present study, it was found that compared with the control, plants treated with IAA were significantly taller, and with the addition of an increased concentration of IAA, the number of stem thorns decreased and the outgrowth time was delayed. Therefore, auxin had an inhibitory effect on the growth of stem thorns of *L. ruthenicum*.

Axillary bud growth is affected by plant hormones, while the role of plant hormones is regulated by related genes. Most early regulated auxin responsive genes are classified into three families: Aux/IAA, Gretchen Hagen3 (GH3), and SAUR (Hagen & Guilfoyle, 2002). Among these genes, *SAURs* are considered to be the most abundant. Since the first *SAUR* gene was identified in elongating soybean hypocotyl sections (McClure & Guilfoyle, 1987), with the availability of genome sequences, genome-wide analysis has been broadly applied, including in the identification and expression profiling of *SAUR* genes in *Arabidopsis* (Hagen & Guilfoyle, 2002), rice (Jain, Tyagi & Khurana, 2006), sorghum (*Glycine max*; Wang et al., 2010), tomato (*Solanum lycopersicum*; Wu et al., 2012), potato (*Solanum tuberosum*; Wu et al., 2012), maize (*Zea mays*; Chen, Hao & Cao, 2014), citrus (Xie et al., 2015), and ramie (*Boehmeria nivea*; Huang et al., 2016). However, there was no reference genome database of *L. ruthenicum* available. In the present study, the SAUR family analysis in *L. ruthenicum* was mainly based on transcriptome data, and a total of 33 possible *SAUR* gene members were identified. Previous reports have indicated that SAURs contain a central domain (PF02519) (Marchler-Bauer et al., 2013), which is highly conserved. The results of the present study confirmed this finding and provided the first systematically identified *SAUR* family members in *L. ruthenicum*.

According to the MEME analysis results, motif 1 and motif 3 are highly conserved in the SAUR family in *L. ruthenicum* (Fig. 3), and may play an important role in the regulation of plant growth and development. The sequence from the N-terminal to the C-terminal was motif 1 and motif 3. However, no motif existed in all family members. Several studies showed that most members of the SAUR proteins have been found to have three highly conserved motifs, RFVIPJSFLSHPLFQDLLSQAEEEFGF, VPKGHFAVYVGE and VPKGHLAVYVG (Wu et al., 2012; Wang et al., 2020; Liu et al., 2022), which were consistent with our results. And RFVIPJSFLSHPLFQDLLSQAEEEFGF was the domain of auxin-inducible superfamily in LrSAURs (Gan, 2019). Kodaira et al. (2011) divided AtSAURs into three classes based on their evolutionary relationships in *A. thaliana*. In the present study, LrSAURs were also divided into three groups (Fig. 2). In addition, evolutionary tree analysis showed that LrSAURs in the same branch had a similar number of motifs and sequences, and there were significant differences among different subgroups (Fig. 2), which may have been due to the acquisition or loss of conserved motifs in the evolutionary process of the SAUR family. Previous studies have shown that stem thorns may be related to drought tolerance in *L. ruthenicum* (Lu, Wang & Fan, 2014; Zhang, 2019), and the absence of *AtSAUR32* in *A. thaliana* may reduce drought tolerance (Kurihara et al., 2021). Guo et al. (2019) found that the overexpression of the *SAUR* gene could enhance tolerance to salt stress, drought stress, and low temperature stress in wheat. Therefore, the expression patterns of *LrSAUR* in response to drought stress were analyzed in combination with transcriptome data in *L. ruthenicum*, and a total of 14 *LrSAURs* were found to have significant changes (Fig. 4). Wang et al. (2012) showed that *SAUR* genes were regulated by IAA and brassinosteroid. Therefore, LrSAURs may play an important role in regulating the growth and development of stem thorns in *L. ruthenicum* under drought conditions. Furthermore, *LrSAUR4*, *LrSAUR5*, *LrSAUR6*, *LrSAUR7*, *LrSAUR8*, *LrSAUR12*, *LrSAUR17*, *LrSAUR19*, *LrSAUR25* and *LrSAUR32* had high expression levels in different stages of stem thorn development (Fig. 4). These results suggest that these genes may be involved in various physiological metabolic processes during the development of *L. ruthenicum*.

This study also investigated the gene expression patterns of 21 *LrSAURs* in various tissues. The number of *LrSAURs* expressed mainly in the leaves was the highest, while the number of genes expressed mainly in the roots was the lowest (Fig. 5), which was similar to previous results in tomato (Wu et al., 2012). In addition, in this study, six *LrSAURs* were highly expressed in stems and thorns (Fig. 5). In cotton, four of 12 *SAURs* were also mainly expressed in the stems (Li et al., 2017). Previous studies have shown that AtSAUR32 is mainly related to apical hook formation in *Arabidopsis* seedlings, and its overexpression causes the apical hook to disappear, while *atsaur32* mutants restore the curved hook phenotype. Notably, *AtSAUR32* is predominantly expressed on the inner side of the apical hook (Park et al., 2007), which provides direct evidence that SAURs function as important regulators of plant accessory structure formation. In the present study, it was found that *LrSAURs* were mainly expressed in stems and thorns, suggesting a putative role in regulating the growth of stem thorns in *L. ruthenicum*. Additionally, compared with the control (C), the expression levels of *LrSAUR9*, *LrSAUR12*, and *LrSAUR19* changed significantly under the D3 treatment (Fig. 6). More study is necessary to further verify the functions of these three *LrSAUR* genes in the development of stem thorns.

##  Supplemental Information

10.7717/peerj.15941/supp-1 Data S1 Raw data of SAUR sequences and expression data

10.7717/peerj.15941/supp-2 Table S1 Primers of Real-time qPCR

## Additional Information and Declarations

### Data Availability

The following information was supplied regarding data availability: The data is available at GenBank: SRR22514555.
