# Supplementary material for: Identification and expression analysis of the small auxin-up RNA (SAUR) gene family in Lycium ruthenicum
Source: PeerJ. 2023 Sep 7;11:e15941. doi: 10.7717/peerj.15941 (PMC10493089; doi:10.7717/peerj.15941)
Supplement: Data S1 [file peerj-11-15941-s001.zip › Raw data/LrSAUR candidate sequences.docx]

>Gene.119965::Lr_transcript_54040::g.119965::m.119965 Gene.119965::Lr_transcript_54040::g.119965 ORF type:complete len:587 (+),score=137.17,tr|Q8GYX8|DNJ10_ARATH|54.63|3e-103,tr|Q8GYX8|DNJ10_ARATH|47.89|3e-73,DnaJ-X|PF14308.5|6.1e-45,DnaJ-X|PF14308.5|4.6e-48,DnaJ|PF00226.30|7.1e-23 Lr_transcript_54040:136-1896(+)

MVKDTEYYDKLGVGVDATPAEIKKAYYFKARTTHPDKNPGDPQAARNFQELGEAYQVLSDPEKRELYDKYGKEDMPKDLMHPAAVFGMLFGSDVFIDYVGELRLASIQSVEDEEDEVVPELRRQNIQEKLKKLQKERVEKLTTILKERLQPYVEGRKDEFLQWAQTEAQHLAQAAFGEAMLHTIGYIYTRQAAKEIGKTKRFMKVPFLAEWVRDKGHLIKSQAMAASGAVSLLQIQEEQKRYQEENKVEDAIKTMEEKKDIMIKSLWQINVVDIELTLSRVCQAVLKEPNVPKDTLRLRARAMKKLGDPEKRELYDKYGKEDMPKDLMHPAAVFGMLFGSDVFIDYVGELRLASIQSVEDEEDEVVPELRRQNIQEKLKKLQKERVEKLTTILKERLQPYVEGRKDEFLQWAQTEAQHLAQAAFGEAMLHTIGYIYTRQAAKEIGKTKRFMKVPFLAEWVRDKGHLIKSQAMAASGAVSLLQIQEEQKRYQEENKVEDAIKTMEEKKDIMIKSLWQINVVDIELTLSRVCQAVLKEPNVPKDTLRLRARAMKKLGTIFQGAKPMYRRESSLRSENIDMVDSGPSSK*

>Gene.115674::Lr_transcript_52466::g.115674::m.115674 Gene.115674::Lr_transcript_52466::g.115674 ORF type:complete len:339 (+),score=77.93,tr|Q8GYX8|DNJ10_ARATH|55.00|1e-109,DnaJ-X|PF14308.5|1.3e-48,DnaJ|PF00226.30|3.1e-23 Lr_transcript_52466:483-1499(+)

MVKDTEYYDKLGVGVDATPAEIKKAYYFKARTTHPDKNPGDPQAARNFQELGEAYQVLSDPEKRELYDKYGKEDMPKDLMHPAAVFGMLFGSDVFIDYVGELRLASIQSVEDEEDEVVPELRRQNIQEKLKKLQKERVEKLTTILKERLQPYVEGRKDEFLQWAQTEAQHLAQAAFGEAMLHTIGYIYTRQAAKEIGKTKRFMKVPFLAEWVRDKGHLIKSQAMAASGAVSLLQIQEEQKRYQEENKVEDAIKTMEEKKDIMIKSLWQINVVDIELTLSRVCQAVLKEPNVPKDTLRLRARAMKKLGTIFQGAKPMYRRESSLRSENIDMVDSGPSSK*

>Gene.31299::Lr_transcript_19866::g.31299::m.31299 Gene.31299::Lr_transcript_19866::g.31299 ORF type:complete len:339 (+),score=76.24,tr|Q8GYX8|DNJ10_ARATH|55.00|1e-109,DnaJ-X|PF14308.5|1.3e-48,DnaJ|PF00226.30|3.1e-23 Lr_transcript_19866:227-1243(+)

MVKDTEYYDKLGVGVDATPAEIKKAYYFKARTTHPDKNPGDPQAARNFQELGEAYQVLSDPEKRELYDKYGKEDMPKDLMHPAAVFGMLFGSDVFIDYVGELRLASIQSVEDEEDEVVPELRRQNIQEKLKKLQKERVEKLTTILKERLQPYVEGRKDEFLQWAQTEAQHLAQAAFGEAMLHTIGYIYTRQAAKEIGKTKRFMKVPFLAEWVRDKGHLIKSQAMAASGAVSLLQIQEEQKRYQEENKVEDAIKTMEEKKDIMIKSLWQINVVDIELTLSRVCQAVLKEPNVPKDTLRLRARAMKKLGTIFQGAKPMYRRESSLRSENIDMVDSGPSSK*

>Gene.27831::Lr_transcript_16978::g.27831::m.27831 Gene.27831::Lr_transcript_16978::g.27831 ORF type:complete len:398 (+),score=103.53,tr|Q8GYX8|DNJ10_ARATH|74.48|0.0,DnaJ-X|PF14308.5|2.7e-51,DnaJ|PF00226.30|3.4e-25 Lr_transcript_16978:273-1466(+)

MVKETEYYDILGVSPTATESEIKKAYYIKARQVHPDKNPNDPQAAQNFQVLGEAYQVLSDPGQRQAYDAHGKSGISTDAIIDPAAIFAMLFGSELFEEYIGQLAMASMASLDIFTEGEDFDAKKLQEKMRVVQREREEKLAETLKDRLNLYVQGNKEEFVRQAEAEVARLSKAAYGVDMLNTIGYIYARQAAKELGKKALFLGLPFIAEWFRNKGHFIKSQVTAATGAIALIQLQEDMKRQLSAEGNYTEEELEEYMQSHKKLMIDSLWKLNVADIEATLSRVCQMVLQDNNVKKEELRARAKGLKTLGKVFQKVKSVDGNETETTANGVHKLDGNEPSFDSRSSTAASAQSPNKEEVPSTVSASQSPYVEAPQFAGGQFSYNFPMPTAPPGAQRHR*

>Gene.124422::Lr_transcript_55641::g.124422::m.124422 Gene.124422::Lr_transcript_55641::g.124422 ORF type:complete len:200 (+),score=53.69,tr|Q8GYX8|DNJ10_ARATH|57.21|7e-67,DnaJ|PF00226.30|1.2e-23,DnaJ-X|PF14308.5|2.7e-18 Lr_transcript_55641:347-946(+)

MVKDTEYYDKLGVGVDATPAEIKKAYYFKARTTHPDKNPGDPQAARNFQELGEAYQVLSDPEKRELYDKYGKEDMPKDLMHPAAVFGMLFGSDVFIDYVGELRLASIQSVEDEEDEVVPELRRQNIQEKLKKLQKERVEKLTTILKERLQPYVEGRKDEFLQWAQTEAQHLAQAAFGEAMLHTIGYIYTRQAAKEIGKT*

>Gene.70413::Lr_transcript_36304::g.70413::m.70413 Gene.70413::Lr_transcript_36304::g.70413 ORF type:complete len:220 (+),score=83.98,tr|Q9XJ56|ECP44_DAUCA|50.24|9e-21,Dehydrin|PF00257.18|1e-15,Dehydrin|PF00257.18|4.6e-07 Lr_transcript_36304:127-786(+)

MADQYEHNKPSGEETGATMVTSDRGLFDKFLGKKEAEKPTHAHEEQAISSEFGEKVRVSEEHKKEEKKEEEHKKEEKKLHRSSSSSSSSSDEEEVIGEDGQKIKKKKKKGLKDKISGDHKEEVKTGKVEDTSVPVEKYEESEEKKGFLDKIKEKLPGGGQKNEEEVAAPAPPPSAVAEHEADVKEKKGFLDKIKEKLPGYHPKTEEEKEKEKEKEAASH*

>Gene.33001::Lr_transcript_21240::g.33001::m.33001 Gene.33001::Lr_transcript_21240::g.33001 ORF type:complete len:229 (+),score=91.99,tr|P31168|COR47_ARATH|40.32|9e-21,Dehydrin|PF00257.18|1.6e-19,Dehydrin|PF00257.18|1.6e-06 Lr_transcript_21240:129-815(+)

MADQYEHNKPSGEETGATMETTDRGLFDKFLGKKEAEKPTHAHEEQAISSEFGEKVKVSEEHKKEEKKEEEHKKEEKKLHRSSSSSSSSSDEEEVIGEDGQKIKKKKKKGLKDKIKDKISGDHKEEVKTGKVEDTSVPVEKYEESDEKKGFLDKIKEKLPGGGQKNEEVVAAPAPPPPAVAEHEADVKEKKGFLDKIKEKLPGYHPKTEEEKEKEKEKEKEKEAAASH*

>Gene.33339::Lr_transcript_21525::g.33339::m.33339 Gene.33339::Lr_transcript_21525::g.33339 ORF type:complete len:225 (+),score=90.66,tr|P31168|COR47_ARATH|40.32|7e-21,Dehydrin|PF00257.18|1.5e-19,Dehydrin|PF00257.18|1.5e-06 Lr_transcript_21525:128-802(+)

MADQYEHNKPSGEETGATMETTDRGLFDKFLGKKEAEKPTHAHEEQAISSEFGEKVKVSEEHKKEEKKEEEHKKEEKKLHRSSSSSSSSSDEEEVIGEDGQKIKKKKKKGLKDKIKDKISGDHKEEVKTGKVEDTSVPVEKYEESDEKKGFLDKIKEKLPGGGQKNEEVVAAPAPPPPAVAEHEADVKEKKGFLDKIKEKLPGYHPKTEEEKEKEKEKEAAASH*

>Gene.170718::Lr_transcript_72535::g.170718::m.170718 Gene.170718::Lr_transcript_72535::g.170718 ORF type:complete len:238 (+),score=100.88,tr|P42759|ERD10_ARATH|36.55|1e-19,Dehydrin|PF00257.18|3.1e-18,Dehydrin|PF00257.18|2.7e-07 Lr_transcript_72535:340-1053(+)

MADQHEHNKTSVEQTGASMESRGLFDFLGKKEEEKPTHVDFLGKKVGEKPTHAHEEQAISSEFGEKVKVSEEEHKKEEKKEEEHRKEEKKLHRSSSSSSSSSDEEEVIGEDGQKIKKKKKKGLKDKIKDKISGDHKEEEVKTEKIEDTSVPVKKYEETEGKKGFLDKIKDKLPGGGQKKEEEVATPATPPPVVAEYEADGKEKKGFLDKIKEKLPGYHPKTEEEKEKEKEKKAAASH*

>Gene.33822::Lr_transcript_21989::g.33822::m.33822 Gene.33822::Lr_transcript_21989::g.33822 ORF type:complete len:199 (+),score=77.97,tr|Q9XJ56|ECP44_DAUCA|44.29|3e-16,Dehydrin|PF00257.18|3.6e-12,Dehydrin|PF00257.18|8.1e-07 Lr_transcript_21989:127-723(+)

MADQYEHNKPSVEETGSTIETKDRGLFDKFLGKKEGEKPTHSHEQQAISSEAGEKVKLSEEHKEEEKKLHRSSSSSSDEEEEVGEDGQKIKKKKKKSLRDIISGEHKEEEKTEKVEDTSVPVEKYEESEEKKGFLDKIKEKLPGDGQKRAEEVAPPPPAVAEHEADGKEKKGLMDKIKEKLPGHHPKTEEEKEAAASY*

>Gene.110887::Lr_transcript_50737::g.110887::m.110887 Gene.110887::Lr_transcript_50737::g.110887 ORF type:complete len:215 (+),score=83.60,tr|Q9XJ56|ECP44_DAUCA|44.81|1e-18,Dehydrin|PF00257.18|2.4e-10,Dehydrin|PF00257.18|9.3e-13 Lr_transcript_50737:128-772(+)

MADQYEHNKPSGEETGATMVTSDRGLFDKFLGKKEAEKPTHAHEEQAISSEFGEKVRVSEEHKKEEKKEEEHKKEEKKESSSSSSDEEEVIGEDGQKIKKKKKKGLKDKISGDHKEEVKTGKVEDTSVPVEKYEESEEKKGFLDKIKEKLPGGGQKNEEEVAAPAPPPSAVAEHEADVKEKKGFLDKIKEKLPGYHPKTEEEKEKEKEKEAASH*

>Gene.34457::Lr_transcript_22575::g.34457::m.34457 Gene.34457::Lr_transcript_22575::g.34457 ORF type:complete len:123 (+),score=15.53,tr|P32295|ARG7_VIGRR|38.04|2e-16,Auxin_inducible|PF02519.13|2.5e-22 Lr_transcript_22575:260-628(+)

MGGQNHHLSFHFHVPLLHFHHHHHGHGKKELKDIPRGCLAITVGQGEEQQRFVIPVIYINHPLFMQLLKEAEEEYGFDHNGPINIPCHIEEFRHVQELIDKETTTHHHNHGHYSHNPWCFKA*

>Gene.35053::Lr_transcript_23176::g.35053::m.35053 Gene.35053::Lr_transcript_23176::g.35053 ORF type:complete len:128 (+),score=29.93,Auxin_inducible|PF02519.13|6.9e-16 Lr_transcript_23176:16-399(+)

MKKMNLLLKKCKTLSRQLGRSSSYSSLRSKSTREDFWNVESQDNKEDYETILVGNSRRRYVIKSKYLSHPLLNALIEKSKQEHGEKDHFSVKCEVVLFDHLLWLLENADPHNLNSDSLEELADLYVV*

>Gene.35062::Lr_transcript_23188::g.35062::m.35062 Gene.35062::Lr_transcript_23188::g.35062 ORF type:complete len:87 (+),score=12.94,tr|P33081|AX15A_SOYBN|69.05|1e-35,Auxin_inducible|PF02519.13|9.7e-26 Lr_transcript_23188:98-358(+)

MAIRMPRIIKNSSTAGDVPKGHFAVYVGEKQKKRFVIPISFLSQPLFQDLLSQAEKEFGFDHPMGGVTIPCSEDVFIDLTSRLNRI*

>Gene.35080::Lr_transcript_23214::g.35080::m.35080 Gene.35080::Lr_transcript_23214::g.35080 ORF type:complete len:86 (+),score=18.15,tr|P33081|AX15A_SOYBN|71.60|1e-35,Auxin_inducible|PF02519.13|6.1e-26 Lr_transcript_23214:96-353(+)

MAIRVPRIIKKSSTSLDVPKGHFAVYVGEKQKKRFVIPISYLSQPSFQDLLSQAEEEFGFDHSMGGVTIPCSEDIFIDITSQFRI*

>Gene.35611::Lr_transcript_23729::g.35611::m.35611 Gene.35611::Lr_transcript_23729::g.35611 ORF type:complete len:87 (+),score=11.50,tr|P33081|AX15A_SOYBN|64.29|3e-32,Auxin_inducible|PF02519.13|1.5e-23 Lr_transcript_23729:8617-8877(+)

MAIRMPRIIKKSSTTGDVPKGHFMVYVGEKLKKRFVIPLSFLSEPLFQDLLSQAEEEFDFNYPLGGLTIPCSEDVFIDLTSQLSRI*

>Gene.43945::Lr_transcript_26687::g.43945::m.43945 Gene.43945::Lr_transcript_26687::g.43945 ORF type:complete len:139 (+),score=29.82,tr|P33083|AX6B_SOYBN|39.71|1e-09,Auxin_inducible|PF02519.13|4.8e-18 Lr_transcript_26687:2698-3114(+)

MMKSTTKMMRKERNNCMLMLRFIMGKLKNHLQLIPKSSRSLEGHVVEFVETPRSNEEVPNDVKEGYFAVFSVNPEEEPKRFIVELHWLTNPSFLKLLKQAEEEYGFEQKGVLEVPCLAADLQKILKLKIGRNITSFAV*

>Gene.60488::Lr_transcript_32697::g.60488::m.60488 Gene.60488::Lr_transcript_32697::g.60488 ORF type:complete len:103 (+),score=9.91,tr|P33081|AX15A_SOYBN|60.71|1e-29,Auxin_inducible|PF02519.13|6.1e-24 Lr_transcript_32697:2359-2667(+)

MHLIIKTMAIHMPRIIKKTSTTGHVPKGHFVVYVGEKQKKRFVIPLSFLSKPLFQDLLSQAEDEFGFNHPLGGLTIPCSEDVLIDFTSQLSRIRGVPFLSFV*

>Gene.88734::Lr_transcript_43000::g.88734::m.88734 Gene.88734::Lr_transcript_43000::g.88734 ORF type:complete len:87 (+),score=13.94,tr|P33081|AX15A_SOYBN|70.24|7e-37,Auxin_inducible|PF02519.13|4.1e-27 Lr_transcript_43000:103-363(+)

MAIRMPRIIKKSSTAGDVPKGHFAVYVGEKQKKRFVIPISFLSQPLFQDLLSQAEEEFGFDHPMGGITIPCSEDVFADLTARLNRI*

>Gene.97395::Lr_transcript_46053::g.97395::m.97395 Gene.97395::Lr_transcript_46053::g.97395 ORF type:complete len:87 (+),score=11.50,tr|P33081|AX15A_SOYBN|64.29|3e-32,Auxin_inducible|PF02519.13|1.5e-23 Lr_transcript_46053:4642-4902(+)

MAIRMPRIIKKSSTTGDVPKGHFMVYVGEKLKKRFVIPLSFLSEPLFQDLLSQAEEEFDFNYPLGGLTIPCSEDVFIDLTSQLSRI*

>Gene.110683::Lr_transcript_50652::g.110683::m.110683 Gene.110683::Lr_transcript_50652::g.110683 ORF type:complete len:149 (+),score=-1.09,tr|P33079|A10A5_SOYBN|45.35|1e-16,Auxin_inducible|PF02519.13|5.2e-28 Lr_transcript_50652:74-520(+)

MSSKMGKSSKIRCIVRISQMLRQWKKRSLISSSKRIAPDVPAGHVAISVGSTCRRFVVRATYLNHPIFRKLLIQAEEEYGFSNHGTLTIPCDELLFEEILRFVSRSGSGRSINIEDFQKSCHARYRNSVENFGDSWPLLGGSTEKSVC*

>Gene.118121::Lr_transcript_53397::g.118121::m.118121 Gene.118121::Lr_transcript_53397::g.118121 ORF type:complete len:134 (+),score=15.85,tr|P33079|A10A5_SOYBN|40.00|4e-07,Auxin_inducible|PF02519.13|1.9e-13 Lr_transcript_53397:183-584(+)

MGSADHKHHHHHLNFHVQVHLPHIHFHHHHHQHGHHGIKELMGIPKGCLPVLVGHDGEELRKFIIPVIYINHPLFTQLLLKGNDLEESELHHDGPINIHCHVEEFRYVEGMIDKETHTTGHHNQHHAAWCYKA*

>Gene.120012::Lr_transcript_54049::g.120012::m.120012 Gene.120012::Lr_transcript_54049::g.120012 ORF type:complete len:139 (+),score=29.82,tr|P33083|AX6B_SOYBN|39.71|1e-09,Auxin_inducible|PF02519.13|4.8e-18 Lr_transcript_54049:65-481(+)

MMKSTTKMMRKERNNCMLMLRFIMGKLKNHLQLIPKSSRSLEGHVVEFVETPRSNEEVPNDVKEGYFAVFSVNPEEEPKRFIVELHWLTNPSFLKLLKQAEEEYGFEQKGVLEVPCLAADLQKILKLKIGRNITSFAV*

>Gene.126716::Lr_transcript_56446::g.126716::m.126716 Gene.126716::Lr_transcript_56446::g.126716 ORF type:complete len:83 (+),score=4.02,tr|P33081|AX15A_SOYBN|56.14|1e-13,Auxin_inducible|PF02519.13|1.9e-10 Lr_transcript_56446:114-362(+)

MAIRMPRIIKKSSTFGDVPKGHLAVYVGEKQNKTYVIPVSFLSQPLFQDLLVKLKKNSVSIIQWVVSQFLAARMCSLISHVA*

>Gene.133486::Lr_transcript_58998::g.133486::m.133486 Gene.133486::Lr_transcript_58998::g.133486 ORF type:complete len:86 (+),score=15.98,tr|P33081|AX15A_SOYBN|64.29|5e-32,Auxin_inducible|PF02519.13|8.8e-25 Lr_transcript_58998:3513-3770(+)

MAIRMPRIIKKSSTAGDVLKGHFAVYVGEKQKKRFVVPLSFLSQPLFQDLLSQAEEEFGFDHPMGGVIIPCNEDFFVDLTSRLRK*

>Gene.137867::Lr_transcript_60669::g.137867::m.137867 Gene.137867::Lr_transcript_60669::g.137867 ORF type:complete len:84 (+),score=21.51,tr|P33081|AX15A_SOYBN|66.67|1e-32,Auxin_inducible|PF02519.13|1e-24 Lr_transcript_60669:4183-4434(+)

MAILRMIKKSSTTRDVPKGHFAVYVGENQKKRFVIPISFLSKPSFQDLLSQAEEEFDFNHPMGGVTIPCSEDLFNDLTSRLRK*

>Gene.142508::Lr_transcript_62376::g.142508::m.142508 Gene.142508::Lr_transcript_62376::g.142508 ORF type:complete len:101 (+),score=15.96,tr|P33083|AX6B_SOYBN|45.59|1e-12,Auxin_inducible|PF02519.13|1.3e-15 Lr_transcript_62376:111-413(+)

MKVKKGWLAIQVGLEEEDGGIQRFVIPISYLYHPLLQKLLDKAHDVYGYHVDGPLKLPCSVDDFLHLRWRIEKEPNRSHHHHHHKNIHQHLPSTLSFHSC*

>Gene.146242::Lr_transcript_63699::g.146242::m.146242 Gene.146242::Lr_transcript_63699::g.146242 ORF type:complete len:96 (+),score=9.36,tr|P33081|AX15A_SOYBN|61.90|4e-30,Auxin_inducible|PF02519.13|4.3e-24 Lr_transcript_63699:3043-3330(+)

MAIHMPRIIKKTSTTGHVPKGHFVVYVGEKQKKRFVIPLSFLSEPLFQDLLSQAEDEFGFNHPMGGLTIPCSEDVLIDFTSQLSRIRGVPFLSFV*

>Gene.172674::Lr_transcript_73228::g.172674::m.172674 Gene.172674::Lr_transcript_73228::g.172674 ORF type:complete len:161 (+),score=26.47,tr|P32295|ARG7_VIGRR|47.54|5e-13,Auxin_inducible|PF02519.13|1.7e-27 Lr_transcript_73228:164-646(+)

MEFDKLCGKSKKGLITKTWKRCTSFGSFGRKNNQQSLSIKSKPWTEGLSTGTGKKNRVVPEGCFSVYVGHQRQRFVIRTKYLNHPLFRMLLEEAESEFGYSSEGPLVLPCDVDIFEKLLMEMDDSDEVDHRRGCSFAAKTHDSYYRLLSPTASAFNKFSF*

>Gene.174128::Lr_transcript_73754::g.174128::m.174128 Gene.174128::Lr_transcript_73754::g.174128 ORF type:complete len:101 (+),score=14.62,tr|P33083|AX6B_SOYBN|47.06|6e-13,Auxin_inducible|PF02519.13|2.1e-15 Lr_transcript_73754:132-434(+)

MKVKKGWLAIQVGLEEEDGGIQRFVIPISYLYHPLLQKLLGKAHDVYGYHVDGPLKLPCSVDDFLHIRWRIEKEPNRSHHHHHHKNIHQHLPSTLSFHSC*

>Gene.193632::Lr_transcript_80939::g.193632::m.193632 Gene.193632::Lr_transcript_80939::g.193632 ORF type:complete len:112 (+),score=20.61,tr|P33083|AX6B_SOYBN|47.44|6e-17,Auxin_inducible|PF02519.13|2.5e-22 Lr_transcript_80939:252-587(+)

MGGGERSLLHLPHLHIHQGKKKTSDVPKGYLAIKVGQEEEEQQRFVVPVSYFNHPLFIQLLKEAEEVYGFHHKGTITIPCHVEQFRSIQGKIDKHHHHNHHHHIHVPCFRA*

>Gene.206924::Lr_transcript_85748::g.206924::m.206924 Gene.206924::Lr_transcript_85748::g.206924 ORF type:complete len:165 (+),score=0.33,tr|P33080|AX10A_SOYBN|44.32|1e-19,Auxin_inducible|PF02519.13|5.6e-24 Lr_transcript_85748:358-852(+)

MRKFRGFVLKHRVTTLFRCIFRRRRWATARYHRLDQLPSWNGPTKSFSRFLNWTQRVKTRAKAICSKAHCYGSGLGYMHVGQDPVEDESVTVPKGHLAVYVGQKDGDYKRVLVPVIYINHPLFSELLREAEEEYGFNHPGGITIPCRISEFEHVQTRIKQGRVG*

>Gene.218255::Lr_transcript_89850::g.218255::m.218255 Gene.218255::Lr_transcript_89850::g.218255 ORF type:complete len:124 (+),score=22.10 Lr_transcript_89850:204-575(+)

MDCIVLPVAILTRRCSGRLLGYRALAEDYGDSDDLITVLVGKEKREFLVEPFVLEESPFRILIEMVRKEDRGRVKENIRTKKVIYVDVDAILFEHMLWLMQNDCSSLFKLNLKEIIEFYAQDI*

>Gene.228330::Lr_transcript_93488::g.228330::m.228330 Gene.228330::Lr_transcript_93488::g.228330 ORF type:complete len:84 (+),score=17.20,tr|P33081|AX15A_SOYBN|65.79|7e-32,Auxin_inducible|PF02519.13|1.4e-24 Lr_transcript_93488:101-352(+)

MAILRLIKKSSTTRDVPKGHFAVYIGETQKKRFVIPISFLSEPLFQDLLSQAEEEFGFDHPMGSVTIPCSEDLFIDLTSRLRN*

>Gene.232877::Lr_transcript_95232::g.232877::m.232877 Gene.232877::Lr_transcript_95232::g.232877 ORF type:complete len:124 (+),score=22.10 Lr_transcript_95232:212-583(+)

MDCIVLPVAILTRRCSGRLLGYRALAEDYGDSDDLITVLVGKEKREFLVEPFVLEESPFRILIEMVRKEDRGRVKENIRTKKVIYVDVDAILFEHMLWLMQNDCSSLFKLNLKEIIEFYAQDI*

>Gene.236195::Lr_transcript_96479::g.236195::m.236195 Gene.236195::Lr_transcript_96479::g.236195 ORF type:complete len:84 (+),score=15.47,tr|P33081|AX15A_SOYBN|63.29|2e-30,Auxin_inducible|PF02519.13|2.2e-25 Lr_transcript_96479:144-395(+)

MAILRMIKKSSTTRDVPKGHFVVYVGETQKKRFVIPISFLSEPLFQKLLSQVEEEFGFDHPMGGVTIPCSEDFFIDLTSRLRK*
