# Supplementary material for: Identification and expression analysis of the small auxin-up RNA (SAUR) gene family in Lycium ruthenicum
Source: PeerJ. 2023 Sep 7;11:e15941. doi: 10.7717/peerj.15941 (PMC10493089; doi:10.7717/peerj.15941)
Supplement: Data S1 [file peerj-11-15941-s001.zip › Raw data/LrSAUR sequences.docx]

>Gene.119965::Lr_transcript_54040

>LrSAUR1

MVKDTEYYDKLGVGVDATPAEIKKAYYFKARTTHPDKNPGDPQAARNFQELGEAYQVLSDPEKRELYDKYGKEDMPKDLMHPAAVFGMLFGSDVFIDYVGELRLASIQSVEDEEDEVVPELRRQNIQEKLKKLQKERVEKLTTILKERLQPYVEGRKDEFLQWAQTEAQHLAQAAFGEAMLHTIGYIYTRQAAKEIGKTKRFMKVPFLAEWVRDKGHLIKSQAMAASGAVSLLQIQEEQKRYQEENKVEDAIKTMEEKKDIMIKSLWQINVVDIELTLSRVCQAVLKEPNVPKDTLRLRARAMKKLGDPEKRELYDKYGKEDMPKDLMHPAAVFGMLFGSDVFIDYVGELRLASIQSVEDEEDEVVPELRRQNIQEKLKKLQKERVEKLTTILKERLQPYVEGRKDEFLQWAQTEAQHLAQAAFGEAMLHTIGYIYTRQAAKEIGKTKRFMKVPFLAEWVRDKGHLIKSQAMAASGAVSLLQIQEEQKRYQEENKVEDAIKTMEEKKDIMIKSLWQINVVDIELTLSRVCQAVLKEPNVPKDTLRLRARAMKKLGTIFQGAKPMYRRESSLRSENIDMVDSGPSSK

>Gene.119965::Lr_transcript_54040::g.119965::m.119965 Gene.119965::Lr_transcript_54040::g.119965 ORF type:complete len:587 (+),score=137.17,tr|Q8GYX8|DNJ10_ARATH|54.63|3e-103,tr|Q8GYX8|DNJ10_ARATH|47.89|3e-73,DnaJ-X|PF14308.5|6.1e-45,DnaJ-X|PF14308.5|4.6e-48,DnaJ|PF00226.30|7.1e-23 Lr_transcript_54040:136-1896(+)

GGAACAAGACCCAATTTAATCCAGCTATTCCAAATTTCTTCTAAATCTGTCCAAAGTTTG

ACCCTTTAATCAAATTTGGCAACATATTGAGTGATTGCAGGTTGTAATCTATTGAAAGTT

TGCATTTGATCAGCTATGGTGAAGGACACAGAGTATTATGACAAATTGGGAGTTGGTGTT

GATGCAACTCCAGCTGAAATTAAGAAAGCTTACTACTTTAAGGCAAGGACTACACATCCA

GACAAGAATCCTGGGGACCCACAAGCCGCTCGTAATTTTCAGGAACTAGGGGAGGCCTAT

CAGGTGCTGAGTGATCCCGAGAAACGTGAACTTTATGACAAATACGGTAAAGAAGATATG

CCGAAGGATTTGATGCACCCTGCAGCTGTATTTGGAATGCTTTTTGGGAGTGATGTATTT

ATTGATTATGTTGGGGAACTTAGATTGGCTTCCATACAATCTGTTGAAGACGAAGAGGAC

GAAGTTGTTCCTGAGCTTCGTAGGCAGAACATTCAGGAGAAATTGAAGAAATTGCAAAAG

GAAAGGGTAGAGAAGCTCACAACAATTCTAAAAGAACGTCTTCAACCATATGTCGAAGGC

CGAAAGGATGAGTTTTTACAATGGGCACAAACAGAAGCGCAACATCTTGCTCAAGCTGCT

TTTGGTGAGGCTATGCTGCATACTATTGGTTATATCTACACAAGGCAAGCTGCAAAAGAA

ATTGGGAAAACTAAAAGATTCATGAAGGTGCCATTTTTGGCAGAATGGGTAAGGGACAAA

GGACACCTAATAAAATCACAAGCGATGGCAGCTTCAGGGGCTGTGTCTTTACTTCAAATA

CAAGAAGAACAGAAGAGGTATCAGGAGGAGAACAAAGTAGAAGATGCAATCAAAACAATG

GAAGAAAAGAAAGATATCATGATTAAATCACTTTGGCAAATTAACGTTGTGGATATTGAG

TTAACTTTATCGCGTGTTTGCCAAGCGGTCCTTAAAGAGCCCAATGTTCCTAAGGATACT

CTCAGGCTACGTGCTAGGGCCATGAAGAAGCTGGGTGATCCCGAGAAACGTGAACTTTAT

GACAAATACGGTAAAGAAGATATGCCGAAGGATTTGATGCACCCTGCAGCTGTATTTGGA

ATGCTTTTTGGGAGTGATGTATTTATTGATTATGTTGGGGAACTTAGATTGGCTTCCATA

CAATCTGTTGAAGACGAAGAGGACGAAGTTGTTCCTGAGCTTCGTAGGCAGAACATTCAG

GAGAAATTGAAGAAATTGCAAAAGGAAAGGGTAGAGAAGCTCACAACAATTCTAAAAGAA

CGTCTTCAACCATATGTCGAAGGCCGAAAGGATGAGTTTTTACAATGGGCACAAACAGAA

GCGCAACATCTTGCTCAAGCTGCTTTTGGTGAGGCTATGCTGCATACTATTGGTTATATC

TACACAAGGCAAGCTGCAAAAGAAATTGGGAAAACTAAAAGATTCATGAAGGTGCCATTT

TTGGCAGAATGGGTAAGGGACAAAGGACACCTAATAAAATCACAAGCGATGGCAGCTTCA

GGGGCTGTGTCTTTACTTCAAATACAAGAAGAACAGAAGAGGTATCAGGAGGAGAACAAA

GTAGAAGATGCAATCAAAACAATGGAAGAAAAGAAAGATATCATGATTAAATCACTTTGG

CAAATTAACGTTGTGGATATTGAGTTAACTTTATCGCGTGTTTGCCAAGCGGTCCTTAAA

GAGCCCAATGTTCCTAAGGATACTCTCAGGCTACGTGCTAGGGCCATGAAGAAGCTGGGT

ACAATTTTTCAGGGTGCGAAGCCAATGTACAGAAGAGAGAGCAGTTTGCGCAGTGAAAAC

ATAGATATGGTAGATAGTGGTCCATCCTCTAAATGAAGCTAATATTTCTCCACTGTATTT

TTTGGTGTAGAGACTTATAACAGCTGCATCCATGCTCGAGACTTGTTCAATGTTGTGCGC

CTCTCTTATTACATTTATGTATCTCCCTGTTTTTCAGACACAGTTTGATCGTTCTATGGC

CAAAATCTCAATTTTTGG

ene.115674::Lr_transcript_52466::g.115674 ORF type:complete len:339 (+),score=77.93,tr|Q8GYX8|DNJ10_ARATH|55.00|1e-109,DnaJ-X|PF14308.5|1.3e-48,DnaJ|PF00226.30|3.1e-23 Lr_transcript_52466:483-1499(+)

GGATTCAAAAGAAAGAAAAAGTTCCACTAAAAAATAACCAATTTGAAGGGATGGGCCGTT

TCATCATCTAAGGCCCATTCATTTTGCACGTGGTTTGTCTGTCGTAAATCAAAGACTCCG

ATCCAAGAACAAAGCCACGTTTTGCATACACTTGTTTATGAATCAAAGAATCTACTTTGT

CATAATCTGTTACACAAGAATTTATGTATCATCTAATTACTTGCTGTTAGCAGAATCCGC

GGTTAAGTTTTCTTTATGTCTGAGCATTGGCGATTGGTTTACGCGTTTAAGCAACTTACA

GCCACACGTTTTAATATTTAAAGATCATCAAGATTTATACATTCATATGAACAAGACCCA

ATTTAATCCAGCTATTCCAAATTTCTTCTAAATCTGTCCAAAGTTTGACCCTTTAATCAA

ATTTGGCAACATATTGAGTGATTGCAGGTTGTAATCTATTGAAAGTTTGCATTTGATCAG

CTATGGTGAAGGACACAGAGTATTATGACAAATTGGGAGTTGGTGTTGATGCAACTCCAG

CTGAAATTAAGAAAGCTTACTACTTTAAGGCAAGGACTACACATCCAGACAAGAATCCTG

GGGACCCACAAGCCGCTCGTAATTTTCAGGAACTAGGGGAGGCCTATCAGGTGCTGAGTG

ATCCCGAGAAACGTGAACTTTATGACAAATACGGTAAAGAAGATATGCCGAAGGATTTGA

TGCACCCTGCAGCTGTATTTGGAATGCTTTTTGGGAGTGATGTATTTATTGATTATGTTG

GGGAACTTAGATTGGCTTCCATACAATCTGTTGAAGACGAAGAGGACGAAGTTGTTCCTG

AGCTTCGTAGGCAGAACATTCAGGAGAAATTGAAGAAATTGCAAAAGGAAAGGGTAGAGA

AGCTCACAACAATTCTAAAAGAACGTCTTCAACCATATGTCGAAGGCCGAAAGGATGAGT

TTTTACAATGGGCACAAACAGAAGCGCAACATCTTGCTCAAGCTGCTTTTGGTGAGGCTA

TGCTGCATACTATTGGTTATATCTACACAAGGCAAGCTGCAAAAGAAATTGGGAAAACTA

AAAGATTCATGAAGGTGCCATTTTTGGCAGAATGGGTAAGGGACAAAGGACACCTAATAA

AATCACAAGCGATGGCAGCTTCAGGGGCTGTGTCTTTACTTCAAATACAAGAAGAACAGA

AGAGGTATCAGGAGGAGAACAAAGTAGAAGATGCAATCAAAACAATGGAAGAAAAGAAAG

ATATCATGATTAAATCACTTTGGCAAATTAACGTTGTGGATATTGAGTTAACTTTATCGC

GTGTTTGCCAAGCGGTCCTTAAAGAGCCCAATGTTCCTAAGGATACTCTCAGGCTACGTG

CTAGGGCCATGAAGAAGCTGGGTACAATTTTTCAGGGTGCGAAGCCAATGTACAGAAGAG

AGAGCAGTTTGCGCAGTGAAAACATAGATATGGTAGATAGTGGTCCATCCTCTAAATGAA

GCTAATATTTCTCCACTGTATTTTTTGGTATAGAGACTTATAACAGCTGCATCCATGCTC

GAGACGTTGTGCGCCTCTCTTATTACATTTGTGTATCTCCCTGTTCTTCAGACACAGTTT

GATCGTTTTATGGCCAAAATCTCAATTTTTGG

>Gene.31299::Lr_transcript_19866

>LrSAUR2

MVKDTEYYDKLGVGVDATPAEIKKAYYFKARTTHPDKNPGDPQAARNFQELGEAYQVLSDPEKRELYDKYGKEDMPKDLMHPAAVFGMLFGSDVFIDYVGELRLASIQSVEDEEDEVVPELRRQNIQEKLKKLQKERVEKLTTILKERLQPYVEGRKDEFLQWAQTEAQHLAQAAFGEAMLHTIGYIYTRQAAKEIGKTKRFMKVPFLAEWVRDKGHLIKSQAMAASGAVSLLQIQEEQKRYQEENKVEDAIKTMEEKKDIMIKSLWQINVVDIELTLSRVCQAVLKEPNVPKDTLRLRARAMKKLGTIFQGAKPMYRRESSLRSENIDMVDSGPSSK

>Gene.27831::Lr_transcript_16978

>LrSAUR3

MVKETEYYDILGVSPTATESEIKKAYYIKARQVHPDKNPNDPQAAQNFQVLGEAYQVLSDPGQRQAYDAHGKSGISTDAIIDPAAIFAMLFGSELFEEYIGQLAMASMASLDIFTEGEDFDAKKLQEKMRVVQREREEKLAETLKDRLNLYVQGNKEEFVRQAEAEVARLSKAAYGVDMLNTIGYIYARQAAKELGKKALFLGLPFIAEWFRNKGHFIKSQVTAATGAIALIQLQEDMKRQLSAEGNYTEEELEEYMQSHKKLMIDSLWKLNVADIEATLSRVCQMVLQDNNVKKEELRARAKGLKTLGKVFQKVKSVDGNETETTANGVHKLDGNEPSFDSRSSTAASAQSPNKEEVPSTVSASQSPYVEAPQFAGGQFSYNFPMPTAPPGAQRHR

Gene.27831::Lr_transcript_16978::g.27831 ORF type:complete len:398 (+),score=103.53,tr|Q8GYX8|DNJ10_ARATH|74.48|0.0,DnaJ-X|PF14308.5|2.7e-51,DnaJ|PF00226.30|3.4e-25 Lr_transcript_16978:273-1466(+)

AACACAGCAATGAATTATCAATTTCCATCTTCCAATTTCATCATCTCTCAAATTTACTAA

TCCTTTCGATCGATTTTTTTTTTTTTTTTTACTTTTGGTTGTTATTTTGTGTTCATTCAT

TCCTTCGTTTTTTATTTTTTCGATCCCCCCCCTTTTTTTTTCCCCAGCTTGAATAGCAGT

TAATTAACCGTACCTTGTGGTTCAGTTTCAAATTTTGTATCTTAATTTCATTTCTATTGA

GAAAGAAAAAAAGTGAACAAAATTAAAGATCGATGGTGAAGGAGACGGAATATTATGACA

TTTTGGGGGTCAGCCCAACTGCTACCGAATCTGAGATTAAAAAAGCTTACTACATTAAGG

CACGGCAAGTTCATCCTGATAAAAACCCAAATGACCCTCAAGCTGCGCAGAATTTTCAGG

TGTTGGGAGAGGCTTATCAAGTACTGAGTGATCCAGGTCAACGACAAGCTTATGATGCAC

ATGGCAAATCAGGAATTTCAACAGATGCAATCATTGATCCTGCAGCAATTTTTGCTATGC

TTTTCGGAAGTGAACTTTTTGAGGAGTACATAGGTCAGCTTGCTATGGCATCAATGGCTT

CATTGGATATTTTCACTGAAGGTGAAGATTTTGATGCAAAAAAATTACAAGAGAAAATGA

GGGTTGTCCAGAGGGAGAGGGAAGAAAAGCTTGCTGAAACTTTGAAAGACCGGCTCAATC

TGTATGTACAAGGAAATAAAGAGGAATTTGTTCGTCAAGCAGAAGCTGAGGTTGCGAGGC

TTTCAAAAGCAGCTTATGGTGTTGATATGTTAAATACAATTGGATACATATATGCAAGGC

AGGCAGCTAAAGAACTCGGGAAGAAGGCATTATTCTTGGGTTTGCCTTTTATTGCTGAGT

GGTTCAGAAACAAAGGCCATTTTATCAAATCTCAAGTAACTGCTGCAACAGGTGCAATTG

CTTTGATTCAGCTACAAGAGGACATGAAAAGGCAGCTCAGCGCAGAAGGAAATTACACCG

AGGAAGAACTTGAAGAGTACATGCAATCCCACAAGAAGCTAATGATTGATTCGCTCTGGA

AGCTTAACGTGGCAGATATTGAAGCCACTCTTTCTCGTGTGTGTCAAATGGTTCTACAAG

ACAACAACGTAAAGAAAGAAGAGCTTCGTGCTCGCGCAAAGGGGTTGAAGACTCTTGGAA

AAGTTTTCCAGAAGGTCAAATCAGTCGATGGAAATGAAACCGAAACTACAGCAAATGGTG

TCCACAAGTTGGATGGAAATGAACCGAGTTTTGACTCTCGTTCTTCAACTGCTGCGTCTG

CACAATCTCCCAATAAAGAAGAGGTGCCTTCTACAGTATCTGCATCACAGAGCCCGTATG

TGGAGGCTCCACAGTTTGCCGGTGGTCAATTCAGCTATAACTTCCCCATGCCGACTGCAC

CACCGGGTGCACAGAGACACAGGTAGACATATGGTGGACGACGCAAGAATTCGCGTAGCT

TGGGAATTGTAGCGCGTGTTGTTGTATGGCTTATTCTTAGGAAAAAGAGAGGGAGTTTGT

ATTTTTTTATATATTATCCCCCGTGTGCGCGTATATTTGAAGCATGATTCCAAGCTTGTG

TAACAGATAGCTGACCAGAGAAAAGCTTCTGTAATATTGATTTCAGGAAGGACTGCTTTG

TAATTAATAGTTCGAGTGTAAGTTGTCTGGTCTGTTTGCATTGGTCTATTCTTGTTCTAT

AACCAATTTAAAGTTTGTTATTGACCTATTCTG

>Gene.124422::Lr_transcript_55641

>LrSAUR4

MVKDTEYYDKLGVGVDATPAEIKKAYYFKARTTHPDKNPGDPQAARNFQELGEAYQVLSDPEKRELYDKYGKEDMPKDLMHPAAVFGMLFGSDVFIDYVGELRLASIQSVEDEEDEVVPELRRQNIQEKLKKLQKERVEKLTTILKERLQPYVEGRKDEFLQWAQTEAQHLAQAAFGEAMLHTIGYIYTRQAAKEIGKT

>Gene.124422::Lr_transcript_55641::g.124422::m.124422 Gene.124422::Lr_transcript_55641::g.124422 ORF type:complete len:200 (+),score=53.69,tr|Q8GYX8|DNJ10_ARATH|57.21|7e-67,DnaJ|PF00226.30|1.2e-23,DnaJ-X|PF14308.5|2.7e-18 Lr_transcript_55641:347-946(+)

GGGTTTTGCATACACTTGTTTATGAATCAAAGAATCTACTTTGTCATAATCTGTTACACA

AGAATTTATGTATCATCTAATTACTTGCTGTTAGCAGAATCCGCGGTTAAGTTTTCTTTA

TGTCTGAGCATTGGCGATTGGTTTACGCGTTTAAGCAACTTACAGCCACACGTTTTAATA

TTTAAAGATCATCAAGATTTATACATTCATATGAACAAGACCCAATTTAATCCAGCTATT

CCAAATTTCTTCTAAATCTGTCCAAAGTTTGACCCTTTAATCAAATTTGGCAACATATTG

AGTGATTGCAGGTTGTAATCTATTGAAAGTTTGCATTTGATCAGCTATGGTGAAGGACAC

AGAGTATTATGACAAATTGGGAGTTGGTGTTGATGCAACTCCAGCTGAAATTAAGAAAGC

TTACTACTTTAAGGCAAGGACTACACATCCAGACAAGAATCCTGGGGACCCACAAGCCGC

TCGTAATTTTCAGGAACTAGGGGAGGCCTATCAGGTGCTGAGTGATCCCGAGAAACGTGA

ACTTTATGACAAATACGGTAAAGAAGATATGCCGAAGGATTTGATGCACCCTGCAGCTGT

ATTTGGAATGCTTTTTGGGAGTGATGTATTTATTGATTATGTTGGGGAACTTAGATTGGC

TTCCATACAATCTGTTGAAGACGAAGAGGACGAAGTTGTTCCTGAGCTTCGTAGGCAGAA

CATTCAGGAGAAATTGAAGAAATTGCAAAAGGAAAGGGTAGAGAAGCTCACAACAATTCT

AAAAGAACGTCTTCAACCATATGTCGAAGGCCGAAAGGATGAGTTTTTACAATGGGCACA

AACAGAAGCGCAACATCTTGCTCAAGCTGCTTTTGGTGAGGCTATGCTGCATACTATTGG

TTATATCTACACAAGGCAAGCTGCAAAAGAAATTGGGAAAACCTAAAAGATTCATGAAGG

TGCCATTTTTGGCAGAATGGGTAAGGGACAAAGGACACCTAATAAAATCACAAGCGATGG

CAGCTTCAGGGGCTGTGTCTTTACTTCAAATACAAGAAGAACAGAAGAGGTATCAGGAGG

AGAACAAAGTAGAAGATGCAATCAAAACAATGGAAGAAAAGAAAGATATCATGATTAAAT

CACTTTGGCAAATTAACGTTGTGGATATTGAGTTAACTTTATCGCGTGTTTGCCAAGCGG

TCCTTAAAGAGCCCAATGTTCCTAAGGATACTCTCAGGCTACGTGCTAGGGCCATGAAGA

AGCTGGGTACAATTTTTCAGGGTGCGAAGCCAATGTACAGAAGAGAGAGCAGTTTGCGCA

GTGAAAACATAGATATGGTAGATAGTGGTCCATCCTCTAAATGAAGCTAATATTTCTCCA

CTGTATTTTTTGGTATAGAGACTTATAACAGCTGCATCCATGCTCGAGACGTTGTGCGCC

TCTCTTATTACATTTGTGTATCTCCCTGTTCTTCAGACACAGTTTGATCGTTTTATGGCC

AAAATCTCAATTTTTGGAAAGCCTTCGACTGAGATTTTGTTGAGAACTTCTATTTATTTG

GGCATAGGTGGTATCTCTTTATTCTTCTTGATATTGACCACATTTCTGATCCATATTTGC

ATTTCAGTGTACTGGTTTGGTATGGGAATAGGGGAACGTGGTGAGATAGGTCAATATCCA

ATCATAGAGCAACTAGTTGCTCGATCTTTGTCTCGGGCTAATCTTGCTTTTTTGAGTGC

>Gene.70413::Lr_transcript_36304

>LrSAUR5

MADQYEHNKPSGEETGATMVTSDRGLFDKFLGKKEAEKPTHAHEEQAISSEFGEKVRVSEEHKKEEKKEEEHKKEEKKLHRSSSSSSSSSDEEEVIGEDGQKIKKKKKKGLKDKISGDHKEEVKTGKVEDTSVPVEKYEESEEKKGFLDKIKEKLPGGGQKNEEEVAAPAPPPSAVAEHEADVKEKKGFLDKIKEKLPGYHPKTEEEKEKEKEKEAASH

>Gene.70413::Lr_transcript_36304::g.70413::m.70413 Gene.70413::Lr_transcript_36304::g.70413 ORF type:complete len:220 (+),score=83.98,tr|Q9XJ56|ECP44_DAUCA|50.24|9e-21,Dehydrin|PF00257.18|1e-15,Dehydrin|PF00257.18|4.6e-07 Lr_transcript_36304:127-786(+)

GACTTCCAATTCAAGTGTCATTTCAACATCCAAAAAGCTTCCTGCTTTAACTTTACCTCA

AAGAGTTATCATTCTTTGCTTTGTTCTTAGCAACAAGTGTTTTTCTGTTTTCTTATTTGA

AAAAAAATGGCTGATCAGTACGAACACAACAAGCCCTCAGGTGAGGAGACTGGTGCCACT

ATGGTGACTTCGGATCGTGGATTGTTTGATAAATTCCTAGGGAAAAAGGAAGCGGAAAAG

CCAACTCATGCTCATGAGGAACAGGCAATTTCCTCTGAGTTTGGTGAAAAAGTAAGGGTA

TCTGAAGAACACAAGAAGGAAGAGAAGAAAGAGGAGGAACATAAGAAAGAAGAGAAGAAA

CTCCACAGGTCAAGTAGCAGCTCTAGCAGCTCGAGTGATGAGGAGGAAGTAATAGGAGAG

GACGGACAGAAAATCAAGAAGAAGAAAAAGAAGGGATTGAAGGACAAAATATCTGGTGAT

CACAAGGAAGAAGTGAAAACAGGGAAGGTTGAGGACACGTCAGTTCCAGTTGAGAAATAC

GAGGAATCGGAGGAGAAAAAAGGATTCCTAGACAAAATTAAGGAGAAGTTGCCAGGTGGC

GGGCAGAAAAATGAGGAGGAAGTGGCGGCGCCTGCACCTCCACCGTCCGCTGTGGCGGAA

CATGAGGCTGATGTGAAAGAGAAGAAGGGATTTTTGGACAAGATTAAGGAGAAATTACCA

GGATACCACCCTAAGACCGAAGAAGAAAAGGAGAAAGAAAAAGAGAAGGAAGCTGCATCT

CACTAAAGTTGATAATAAAACAAGCAAATGAATGTTTATTTGTTGTTTTTTACTTTTGGC

GTTTTGGGATGTGTTATGATTGTGTTGGTTAAGCTTTTGTTCATTTGCTCCACTTTTTGG

ATTCTGAAAGATATTATTACTGTATTCTTGTGTGTAAGTGCATAGGTTTTCATGTGCTTA

TGTCTAAAGCTTTGACGATATGGATTTGTAAAATGCAGTTGTTTTGTTTTATGACTTCCT

GTTTGTTTTGGAATTTAGTTGTATTGTATTATAAAAGCTACTGTATAATGTATATTTTTA

TTTT

>Gene.33001::Lr_transcript_21240

>LrSAUR6

MADQYEHNKPSGEETGATMETTDRGLFDKFLGKKEAEKPTHAHEEQAISSEFGEKVKVSEEHKKEEKKEEEHKKEEKKLHRSSSSSSSSSDEEEVIGEDGQKIKKKKKKGLKDKIKDKISGDHKEEVKTGKVEDTSVPVEKYEESDEKKGFLDKIKEKLPGGGQKNEEVVAAPAPPPPAVAEHEADVKEKKGFLDKIKEKLPGYHPKTEEEKEKEKEKEKEKEAAASH

Gene.33001::Lr_transcript_21240::g.33001 ORF type:complete len:229 (+),score=91.99,tr|P31168|COR47_ARATH|40.32|9e-21,Dehydrin|PF00257.18|1.6e-19,Dehydrin|PF00257.18|1.6e-06 Lr_transcript_21240:129-815(+)

GCACTTCCAATTCAAGTGTCATTTCAACATCCAAAAAGCTTCCTGCTTTAACTTTACCTC

AAAGAGTTATCATTCTTTGCTTTGTTCTTAGCAACAAGTGTTTTTTTGTTTTTCTTATTT

GAAAAAAAATGGCTGATCAGTACGAACACAACAAGCCCTCAGGTGAGGAGACTGGTGCCA

CTATGGAGACTACGGATCGTGGATTGTTTGATAAATTCTTAGGGAAAAAGGAAGCGGAAA

AGCCAACTCATGCTCATGAGGAACAGGCAATTTCCTCTGAGTTTGGTGAAAAAGTAAAGG

TATCTGAAGAACACAAGAAGGAAGAGAAGAAAGAGGAGGAACATAAGAAAGAAGAGAAGA

AACTCCACAGGTCAAGTAGCAGCTCTAGCAGCTCGAGTGATGAGGAGGAAGTAATAGGAG

AGGACGGACAGAAAATCAAGAAGAAGAAAAAGAAGGGATTGAAGGACAAGATCAAGGACA

AAATATCTGGTGATCACAAGGAAGAAGTGAAAACAGGGAAGGTTGAGGACACGTCAGTTC

CAGTTGAGAAATACGAGGAATCGGATGAGAAAAAAGGATTCCTAGACAAAATTAAGGAGA

AGTTGCCAGGTGGCGGGCAGAAAAACGAGGAGGTAGTGGCGGCGCCTGCACCTCCACCAC

CTGCTGTGGCGGAACATGAGGCTGATGTGAAAGAGAAGAAGGGATTTTTGGACAAGATTA

AGGAGAAATTACCAGGATATCACCCCAAGACCGAAGAAGAAAAGGAGAAAGAAAAAGAGA

AAGAAAAAGAGAAGGAAGCTGCTGCATCCCACTAAAGTTGATACTAAAGTTGATAAAACA

AGCAAATGAATGTTTATTTGTTGTTTTTTACTTTTGGCGTTTTGGGGTGTGTTATGATTG

TGTTGGTTAAGCTTTTGTTCACTTGCTCCACTGTTGTTTTGGATTCTGAAAGATATTATT

ACTGTATTCTTGTGTGTAAGTGCATAGGTTTTCATGTGCTTATGATGTCTAAAGCTTTGA

TGACATGGATTTGTAAAATGCAGTTGTTTTGTTTTATGACTTCCTGTTTGTTTTGGATTT

TAGTTGTATTGTATTATAAAAGCTACTGTATAATGTATATTTAATTTTAAATTT

>Gene.33339::Lr_transcript_21525

>LrSAUR7

MADQYEHNKPSGEETGATMETTDRGLFDKFLGKKEAEKPTHAHEEQAISSEFGEKVKVSEEHKKEEKKEEEHKKEEKKLHRSSSSSSSSSDEEEVIGEDGQKIKKKKKKGLKDKIKDKISGDHKEEVKTGKVEDTSVPVEKYEESDEKKGFLDKIKEKLPGGGQKNEEVVAAPAPPPPAVAEHEADVKEKKGFLDKIKEKLPGYHPKTEEEKEKEKEKEAAASH

T

>Gene.33339::Lr_transcript_21525::g.33339::m.33339 Gene.33339::Lr_transcript_21525::g.33339 ORF type:complete len:225 (+),score=90.66,tr|P31168|COR47_ARATH|40.32|7e-21,Dehydrin|PF00257.18|1.5e-19,Dehydrin|PF00257.18|1.5e-06 Lr_transcript_21525:128-802(+)

GACTTCCAATTCAAGTGTCATTTCAACATCCAAAAAGCTTCCTGCTTTAACTTTACCTCA

AAGAGTTATCATTCTTTGCTTTGTTCTTAGCAACAAGTGTTTTTTTGTTTTTCTTATTTG

AAAAAAAATGGCTGATCAGTACGAACACAACAAGCCCTCAGGTGAGGAGACTGGTGCCAC

TATGGAGACTACGGATCGTGGATTGTTTGATAAATTCTTAGGGAAAAAGGAAGCGGAAAA

GCCAACTCATGCTCATGAGGAACAGGCAATTTCCTCTGAGTTTGGTGAAAAAGTAAAGGT

ATCTGAAGAACACAAGAAGGAAGAGAAGAAAGAGGAGGAACATAAGAAAGAAGAGAAGAA

ACTCCACAGGTCAAGTAGCAGCTCTAGCAGCTCGAGTGATGAGGAGGAAGTAATAGGAGA

GGACGGACAGAAAATCAAGAAGAAGAAAAAGAAGGGATTGAAGGACAAGATCAAGGACAA

AATATCTGGTGATCACAAGGAAGAAGTGAAAACAGGGAAGGTTGAGGACACGTCAGTTCC

AGTTGAGAAATACGAGGAATCTGATGAGAAAAAAGGATTCCTAGACAAAATTAAGGAGAA

GTTGCCAGGTGGCGGGCAGAAAAACGAGGAGGTAGTGGCGGCGCCTGCACCTCCACCACC

TGCTGTGGCGGAACATGAGGCTGATGTGAAAGAGAAGAAGGGATTTTTGGACAAGATTAA

GGAGAAATTACCAGGATATCACCCCAAGACCGAAGAAGAAAAGGAGAAAGAAAAAGAGAA

GGAAGCTGCTGCATCCCACTAAAGTTGATACTAAAGTTGATAAAACAAGCAAATGAATGT

TTATTTGTTGTTTTTTACTTTTGGCGTTTTGGGGTGTGTTATGATTGTGTTGGTTAAGCT

TTTGTTCACTTGCTCCACTGTTGTTTTGGATTCTGAAAGATATTATTACTGTATTCTTGT

GTGTAAGTGCATAGGTTTTCATGTGCTTATGATGTCTAAAGCTTTGATGACATGGATTTG

TAAAATGCAGTTGTTTTGTTTT

>Gene.170718::Lr_transcript_72535

>LrSAUR8

MADQHEHNKTSVEQTGASMESRGLFDFLGKKEEEKPTHVDFLGKKVGEKPTHAHEEQAISSEFGEKVKVSEEEHKKEEKKEEEHRKEEKKLHRSSSSSSSSSDEEEVIGEDGQKIKKKKKKGLKDKIKDKISGDHKEEEVKTEKIEDTSVPVKKYEETEGKKGFLDKIKDKLPGGGQKKEEEVATPATPPPVVAEYEADGKEKKGFLDKIKEKLPGYHPKTEEEKEKEKEKKAAASH

>Gene.170718::Lr_transcript_72535::g.170718::m.170718 Gene.170718::Lr_transcript_72535::g.170718 ORF type:complete len:238 (+),score=100.88,tr|P42759|ERD10_ARATH|36.55|1e-19,Dehydrin|PF00257.18|3.1e-18,Dehydrin|PF00257.18|2.7e-07 Lr_transcript_72535:340-1053(+)

GATATCAAAAAAGCTTCAAACTTTCACTTTACCTCTAAGAGCCCGTTTGGATTGGCTTAT

AAGCTGTTTTTAATTTTTTTTAGTGTTTAGCTGCCTAGCTTAAAGTCATTTTGTATTTAA

AATAAGCTCAAAAAAATAATTGAGTTCATTTGACTTAACTTATCTAAAGCAACTTATAAG

CTGAAAATAATTTATAAGCAAAAAAAAAATAAGTTGAACTACCAATTTTTTTTTTAATTT

ATAAGCTGTTTTCAGCTTAAAGTCATAAGCCCATCCAAACAGGCTCTAAAGAGTGGTCAT

TCTTTGTAACAAGTTTCTCTTTGTTTTTGTTTGAAAAAAATGGCTGATCAGCACGAACAC

AACAAGACCTCAGTTGAACAGACTGGTGCCAGCATGGAGTCTCGTGGTTTGTTTGATTTC

CTTGGGAAAAAAGAAGAGGAAAAGCCAACTCATGTTGATTTCCTCGGGAAAAAAGTAGGG

GAAAAGCCAACTCATGCTCATGAGGAACAGGCAATTTCCTCTGAATTTGGTGAGAAAGTA

AAGGTATCTGAAGAAGAACACAAGAAGGAAGAGAAGAAAGAGGAGGAACACAGGAAAGAA

GAGAAGAAACTCCACAGATCAAGTAGCAGCTCTAGCAGCTCGAGTGATGAGGAGGAAGTA

ATAGGAGAGGATGGACAAAAAATCAAGAAGAAGAAAAAGAAGGGGTTGAAGGACAAGATC

AAGGACAAAATATCTGGTGATCACAAGGAAGAAGAAGTGAAAACAGAGAAGATTGAGGAC

ACGTCAGTTCCAGTTAAGAAGTATGAGGAAACAGAGGGAAAAAAAGGTTTCCTAGATAAA

ATCAAGGACAAGTTGCCAGGTGGCGGGCAGAAGAAGGAGGAGGAAGTGGCGACGCCAGCA

ACTCCACCACCAGTTGTGGCGGAGTATGAGGCTGATGGTAAGGAGAAGAAGGGATTTTTG

GACAAAATTAAGGAGAAATTACCAGGATACCACCCTAAGACTGAAGAAGAAAAGGAGAAA

GAAAAAGAAAAGAAAGCTGCCGCATCTCACTAAAGTTGGCAGTATGGGAATGGATGTTTA

TGTTTATTTGTTGCTTTTTTGCTTTTGAGATGTTTTGATTGTTCTGCTATGCTTTGGTTA

AGCTTTCATTTCAATTCCTTCGCTGTTTCTGGATTTTTAGGACTGTATTCCTATATGCAA

GTGCATAGCTGTTTTGTATGTGCTCATAGTTGTTTTGTATGTGCTTATGTATAAAGTTTG

ATTATATATGGATTTGTAAAATGCAGTTGGTTTTGATT

>Gene.33822::Lr_transcript_21989

>LrSAUR9

MADQYEHNKPSVEETGSTIETKDRGLFDKFLGKKEGEKPTHSHEQQAISSEAGEKVKLSEEHKEEEKKLHRSSSSSSDEEEEVGEDGQKIKKKKKKSLRDIISGEHKEEEKTEKVEDTSVPVEKYEESEEKKGFLDKIKEKLPGDGQKRAEEVAPPPPAVAEHEADGKEKKGLMDKIKEKLPGHHPKTEEEKEAAASY

Gene.33822::Lr_transcript_21989::g.33822 ORF type:complete len:199 (+),score=77.97,tr|Q9XJ56|ECP44_DAUCA|44.29|3e-16,Dehydrin|PF00257.18|3.6e-12,Dehydrin|PF00257.18|8.1e-07 Lr_transcript_21989:127-723(+)

GATCCAATTTAAGTTTCATTTCAATATCCAAAAAGCTTCGAACTTTCACTTTACCTCAAG

GAGTTTTCGTTCTTTGCTTTGTTCTTAGCAACAAGTTTTTATTTGTTTTTGTTATTTGCA

AAAAAAATGGCTGATCAGTACGAACACAACAAGCCCTCAGTTGAAGAGACTGGTTCCACT

ATAGAGACTAAAGATCGTGGTTTGTTTGATAAATTCCTAGGGAAAAAGGAAGGGGAAAAG

CCAACTCATTCTCATGAACAACAGGCAATTTCCTCTGAGGCTGGTGAGAAAGTAAAATTA

TCTGAAGAACACAAGGAGGAAGAGAAGAAACTCCACAGATCAAGTAGTAGCTCGAGTGAT

GAGGAAGAAGAAGTAGGAGAGGACGGACAGAAAATCAAGAAGAAGAAAAAGAAGAGCTTG

AGGGACATAATATCTGGAGAGCACAAGGAAGAAGAGAAAACGGAGAAGGTTGAGGACACG

TCAGTTCCTGTTGAGAAATATGAGGAATCCGAGGAGAAAAAAGGTTTCCTAGACAAAATA

AAGGAGAAGTTGCCAGGTGACGGGCAGAAGAGAGCTGAGGAAGTGGCGCCTCCACCACCA

GCTGTGGCGGAGCATGAGGCTGATGGTAAGGAAAAGAAGGGATTAATGGACAAAATTAAG

GAGAAATTACCGGGACACCACCCTAAGACTGAAGAAGAAAAGGAAGCTGCTGCCTCTTAC

TAAAGTTGACAATATGGGAATGAATAAAGTGTTTATTTGTGTCTTTTTTACTTTTATGAT

GTTGTGATTGTGCCTGCTATATGATGTTTTGTTTAAGCTTTCATTTCAATTCCTGCACTG

TTTCTGGATTTTAGGAGGACTGTATTCCTCTATGTCAGTACATAGTTGTTTTGTATGTGC

TTATATATAAAGTTTGTTATATATGGATTTGTAAAATGCAGTTGGCTTTG

>Gene.110887::Lr_transcript_50737

>LrSAUR10

MADQYEHNKPSGEETGATMVTSDRGLFDKFLGKKEAEKPTHAHEEQAISSEFGEKVRVSEEHKKEEKKEEEHKKEEKKESSSSSSDEEEVIGEDGQKIKKKKKKGLKDKISGDHKEEVKTGKVEDTSVPVEKYEESEEKKGFLDKIKEKLPGGGQKNEEEVAAPAPPPSAVAEHEADVKEKKGFLDKIKEKLPGYHPKTEEEKEKEKEKEAASH

>Gene.110887::Lr_transcript_50737::g.110887::m.110887 Gene.110887::Lr_transcript_50737::g.110887 ORF type:complete len:215 (+),score=83.60,tr|Q9XJ56|ECP44_DAUCA|44.81|1e-18,Dehydrin|PF00257.18|2.4e-10,Dehydrin|PF00257.18|9.3e-13 Lr_transcript_50737:128-772(+)

GACTTCCAATTCAAGTGTCATTTCAACATCCAAAAAGCTTCCTGCTTTAACTTTACCTCA

AAGAGTTATCATTCTTTGCTTTGTTCTTAGCAACAAGTGTTTTTCTGTTTTTCTTATTTG

AAAAAAAATGGCTGATCAGTACGAACACAACAAGCCCTCAGGTGAGGAGACTGGTGCCAC

TATGGTGACTTCGGATCGTGGATTGTTTGATAAATTCCTAGGGAAAAAGGAAGCGGAAAA

GCCAACTCATGCTCATGAGGAACAGGCAATTTCCTCTGAGTTTGGTGAAAAAGTAAGGGT

ATCTGAAGAACACAAGAAGGAAGAGAAGAAAGAGGAGGAACATAAGAAAGAAGAGAAGAA

AGAGAGCTCTAGCAGCTCGAGTGATGAGGAGGAAGTAATAGGAGAGGACGGACAGAAAAT

CAAGAAGAAGAAAAAGAAGGGATTGAAGGACAAAATATCTGGTGATCACAAGGAAGAAGT

GAAAACAGGGAAGGTTGAGGACACGTCAGTTCCAGTTGAGAAATACGAGGAATCGGAGGA

GAAAAAAGGATTCCTGGACAAAATTAAGGAGAAGTTGCCAGGTGGCGGGCAGAAAAATGA

GGAGGAAGTGGCGGCGCCTGCACCTCCACCGTCCGCTGTGGCGGAACATGAGGCTGATGT

GAAAGAGAAGAAGGGATTTTTGGACAAGATTAAGGAGAAATTACCAGGATACCACCCTAA

GACCGAAGAAGAAAAGGAGAAAGAAAAAGAGAAGGAAGCTGCATCTCACTAAAGTTGATA

ATAAAACAAGCAAATGAATGTTTATTTGTTGTTTTTTACTTTTGGCGTTTTGGGATGTGT

TATGATTGTGTTGGTTAAGCTTTTGTTCATTTGCTCCACTTTTTGGATTCTGAAAGATAT

TATTACTGTATTCTTGTGTGTAAGTGCATAGGTTTTCATGTGCTTATGTCTAAAGCTTTG

ATGATATGGATTTGTAAAATGCAGTTGTTTTGTCG

>Gene.34457::Lr_transcript_22575

>LrSAUR11

MGGQNHHLSFHFHVPLLHFHHHHHGHGKKELKDIPRGCLAITVGQGEEQQRFVIPVIYINHPLFMQLLKEAEEEYGFDHNGPINIPCHIEEFRHVQELIDKETTTHHHNHGHYSHNPWCFKA

>Gene.110887::Lr_transcript_50737::g.110887::m.110887 Gene.110887::Lr_transcript_50737::g.110887 ORF type:complete len:215 (+),score=83.60,tr|Q9XJ56|ECP44_DAUCA|44.81|1e-18,Dehydrin|PF00257.18|2.4e-10,Dehydrin|PF00257.18|9.3e-13 Lr_transcript_50737:128-772(+)

GACTTCCAATTCAAGTGTCATTTCAACATCCAAAAAGCTTCCTGCTTTAACTTTACCTCA

AAGAGTTATCATTCTTTGCTTTGTTCTTAGCAACAAGTGTTTTTCTGTTTTTCTTATTTG

AAAAAAAATGGCTGATCAGTACGAACACAACAAGCCCTCAGGTGAGGAGACTGGTGCCAC

TATGGTGACTTCGGATCGTGGATTGTTTGATAAATTCCTAGGGAAAAAGGAAGCGGAAAA

GCCAACTCATGCTCATGAGGAACAGGCAATTTCCTCTGAGTTTGGTGAAAAAGTAAGGGT

ATCTGAAGAACACAAGAAGGAAGAGAAGAAAGAGGAGGAACATAAGAAAGAAGAGAAGAA

AGAGAGCTCTAGCAGCTCGAGTGATGAGGAGGAAGTAATAGGAGAGGACGGACAGAAAAT

CAAGAAGAAGAAAAAGAAGGGATTGAAGGACAAAATATCTGGTGATCACAAGGAAGAAGT

GAAAACAGGGAAGGTTGAGGACACGTCAGTTCCAGTTGAGAAATACGAGGAATCGGAGGA

GAAAAAAGGATTCCTGGACAAAATTAAGGAGAAGTTGCCAGGTGGCGGGCAGAAAAATGA

GGAGGAAGTGGCGGCGCCTGCACCTCCACCGTCCGCTGTGGCGGAACATGAGGCTGATGT

GAAAGAGAAGAAGGGATTTTTGGACAAGATTAAGGAGAAATTACCAGGATACCACCCTAA

GACCGAAGAAGAAAAGGAGAAAGAAAAAGAGAAGGAAGCTGCATCTCACTAAAGTTGATA

ATAAAACAAGCAAATGAATGTTTATTTGTTGTTTTTTACTTTTGGCGTTTTGGGATGTGT

TATGATTGTGTTGGTTAAGCTTTTGTTCATTTGCTCCACTTTTTGGATTCTGAAAGATAT

TATTACTGTATTCTTGTGTGTAAGTGCATAGGTTTTCATGTGCTTATGTCTAAAGCTTTG

ATGATATGGATTTGTAAAATGCAGTTGTTTTGTCG

>Gene.35053::Lr_transcript_23176

>LrSAUR12

MKKMNLLLKKCKTLSRQLGRSSSYSSLRSKSTREDFWNVESQDNKEDYETILVGNSRRRYVIKSKYLSHPLLNALIEKSKQEHGEKDHFSVKCEVVLFDHLLWLLENADPHNLNSDSLEELADLYVV

Gene.35053::Lr_transcript_23176::g.35053 ORF type:complete len:128 (+),score=29.93,Auxin_inducible|PF02519.13|6.9e-16 Lr_transcript_23176:16-399(+)

GGGAATTAAGAGAAGATGAAGAAGATGAATCTGTTACTGAAGAAGTGTAAGACTTTGTCA

AGACAACTAGGAAGAAGCTCATCTTATAGTAGTTTAAGGTCAAAATCTACAAGAGAAGAT

TTTTGGAATGTGGAATCTCAAGATAATAAAGAGGATTATGAGACTATTCTTGTTGGCAAC

TCAAGAAGGAGATATGTGATAAAATCCAAATATTTGAGCCATCCACTATTGAATGCTCTA

ATAGAGAAATCAAAGCAGGAACATGGGGAGAAAGATCATTTTTCAGTTAAGTGTGAAGTT

GTGCTTTTTGATCATCTTCTCTGGTTGCTTGAAAATGCTGACCCCCACAACCTGAATTCT

GATTCTTTGGAGGAATTGGCTGATCTATATGTTGTTTAATTTGAATATTATTCAATAATA

TTCAATCTTCCTTTTCATTCTCTAGTCAAAAATATGTTTTAGCTCTAGTATTTTTTCATG

TAACTAGTAGTAATAAATGTTAAGGAGTGACTATCTCTCATAAAAATGAGAAGGATCATA

GTTTCCTATT

>Gene.35062::Lr_transcript_23188

>LrSAUR13

MAIRMPRIIKNSSTAGDVPKGHFAVYVGEKQKKRFVIPISFLSQPLFQDLLSQAEKEFGFDHPMGGVTIPCSEDVFIDLTSRLNRI

Gene.35062::Lr_transcript_23188::g.35062 ORF type:complete len:87 (+),score=12.94,tr|P33081|AX15A_SOYBN|69.05|1e-35,Auxin_inducible|PF02519.13|9.7e-26 Lr_transcript_23188:98-358(+)

GGATAGCAGCTCTCACCAAAACTTGAAATCATACAAAGCTTTCCTCTGGTTTCAAAGTCC

TTCCCTTATTTGATTATTTATATTGAACTAAACGACCATGGCTATCCGTATGCCTCGTAT

AATCAAGAACTCATCTACGGCTGGAGACGTTCCGAAAGGCCACTTTGCTGTTTATGTTGG

GGAGAAGCAAAAGAAGAGATTTGTAATCCCCATATCTTTCTTGAGCCAACCATTATTTCA

AGACTTGCTTAGTCAAGCTGAGAAAGAATTTGGCTTTGATCATCCAATGGGCGGTGTCAC

AATTCCATGTAGCGAGGATGTGTTCATTGATCTTACCTCCCGCTTGAATAGGATCTGAGG

ATTTTCCTTTCACAATTTTGTAAGGAACTAATCTGGATTTCCAGTTTGTACATTAGAAGA

GTTAGGAAAGACACAATTTTGTATCATAACTTAGAAAATTAGATAGAGGGGACTACTTTC

AGATGCAACAGTAGATCCCAGTTGAATGTAATTATCAGCACTTTAGAAAATGAATTCTCT

TCAGACTC

>Gene.35080::Lr_transcript_23214

>LrSAUR14

MAIRVPRIIKKSSTSLDVPKGHFAVYVGEKQKKRFVIPISYLSQPSFQDLLSQAEEEFGFDHSMGGVTIPCSEDIFIDITSQFRI

>Gene.35611::Lr_transcript_23729

>LrSAUR15

MAIRMPRIIKKSSTTGDVPKGHFMVYVGEKLKKRFVIPLSFLSEPLFQDLLSQAEEEFDFNYPLGGLTIPCSEDVFIDLTSQLSRI

>Gene.43945::Lr_transcript_26687

>LrSAUR16

MMKSTTKMMRKERNNCMLMLRFIMGKLKNHLQLIPKSSRSLEGHVVEFVETPRSNEEVPNDVKEGYFAVFSVNPEEEPKRFIVELHWLTNPSFLKLLKQAEEEYGFEQKGVLEVPCLAADLQKILKLKIGRNITSFAV

>Gene.60488::Lr_transcript_32697

>LrSAUR17

MHLIIKTMAIHMPRIIKKTSTTGHVPKGHFVVYVGEKQKKRFVIPLSFLSKPLFQDLLSQAEDEFGFNHPLGGLTIPCSEDVLIDFTSQLSRIRGVPFLSFV

>Gene.60488::Lr_transcript_32697::g.60488::m.60488 Gene.60488::Lr_transcript_32697::g.60488 ORF type:complete len:103 (+),score=9.91,tr|P33081|AX15A_SOYBN|60.71|1e-29,Auxin_inducible|PF02519.13|6.1e-24 Lr_transcript_32697:2359-2667(+)

ACACACTCAAAAGAAGAAAACACTCTTTTGATTTTTCCCACCTCACTACAATATCACTCA

CATTCTATTTTTCTTCCCAGACTATTTTCTTACCGTCTATGGAATACCTCACTTTGCTCT

CACTCAGATGTATTGTCTGAGATTTTTGGTGTGTATAGCAAATGATCTTGGTGATCATAC

AAATGAACCATAAGCTGCCTATTTATAGGAATGAATTTCCTATGATTAGGTAAGCGCTTA

CATCACGAATATGATGAGGTAAGCGCTTACATACGACTAAAATGAGGTAAGCGATTACAT

CACGACTCTGTGAACAAAAGAATTGACTTGTTGGTCAATTACACAATTGCTACCAACGTG

ATTTTGTCAAATTTCATTCGTTAAAAATAGAGATAGGAGACTGGACGCCACATGACTTTC

ATATCAAACTCATATGATATCTACATGAACAAATTTACGTTTCCTAACAACAGCTTCTTC

AAGATTTTCAACACCCAAAAAAAAAAAAAACTTTGCTATTGAATTCAATATTTTTCCCAT

TATTTACAAAATGGGTTACATGCTTTTGCTCTCCATCACTTGTCTTCCATGAATTCAATT

AACAGTGTTCGCATTGATCACTACAGGTATATGTCTGTAACATATCTTCATCTTGTCTTC

AAAACATGAACTTTGGTTCAAGTGATTCACGCCCTTTTACATGAATATTCTGGTTTTCTT

TTAAGCGTATATTTGTGCTCTGCTTTGGTTGATTTGTATGCCTAGTCATTGTTTACCAAG

AATTCCCCTGAGTTGTTCGATGAAATTCCTGTGAAGAAGAATGCTGTATGTGCCAAATGC

CATTTTTAAACCTGTACACGGATTGGGTATTTCTCTCTTTTATCTGGATTGGGTAAGCAG

GTTCAGGGATGTGTTATTGGAAAAGCTCATGGCATGGAAACTGATCAAATTATGTTGTTT

GAATATTATGGCAAATGTGGACTAATGGCCCCATGCCCTACTACAATTCTTATTCAATAT

AATACTTCCTCCGTCCCAATTTAAGTGTCTTATTTTTCTTTTTAGTCTATCCCAAAAAGA

AGGCCTCTTTCTCTATTTGGTAAGTTTTCCAATCCCAAGACAAGTTTAAGACTATAAGAT

TTATAAGACTACACATATCTTTTATTTAAGACCACAAGATTCAAAAATATTCCTTTATTT

CTTAAACTCCGTGCTCAGTCAAACTAGGACACTTAAATTGGGACGGACATTACTACAATG

TCGTGCCGATGTCTCCCTTACGAAATTGAAGATAAGTCTTTTGATTAGGGGGGGTGACAA

CCGAGTTGTTTAAGAGAAGTTGCCATAGCGGCTATAACGAGCTATCTCATATAAGGCTAG

GCTTGTATCCAATAAATTTAGAGTAGTGATTAGTTTGTTTAAGGGCTTGGTAGCGGAGGG

AACTAAACCAGATGGTGTAGTAAGTCTTGGGATACGAATTTTGATTAAGTATGTGATACA

CTGTTCCGAGCATTATAATTGTTTAATAGACTTTATTTGTCATGCCAGTGTACAACAATG

CCTCAATCCCAGGTAAGTTGTAGTCGGCTATATGAATCCTCATTGACTTTGCCGATCCAT

TAAAGCTTATGGTAGACTCATTGTCATGCCAGTGTATGGAAATTAGTTAGCAATTTCTCC

TCTAATAAGAATGACAAGTTACTGTTTCTATGTAGGAGGTCTTACTGAGTTCCTGAAATT

ATTTTGACAATGTTGAGTCATTAAAGGGTACTCCAGTTGGACCCTCTAAATAACATGGTT

TAAGTTTTTGCTTTCAAAATGTATGCAAGGAATAGTATGTGGAATGAGAATGAGAAACCG

AGGTAACAGAGAACAGCCTAAAGAAAGGCAGTGACATAGCGTGGTAGACGTCACAAGATG

AGTTAAAGATGTTGATATTAAAGAAACCACATTCTCCTACTCATGGATTCATGTTGGTCA

GTCGAAAATGGTTTGAGGTACAGTGGTGCAGTTATAGGACTTTGGTCGCTTGTGGTATAT

TTCGAAAAATGTGATAAGGGACTCTTCTTGATGGAGTTGGAATGTTCATAATGAGGTCTC

TTACACGGAAGTGCTGTCACAAGTTTGAATTTCTGTAAGATTGAGCAAACTGCAAGACAA

TGCTTCACCATGCCGTAGTTTCTTCGCATAGAATATGAGAGCAGTAACAGGCTGAGAACA

AATGAATTTCTTAACAGTGCAAAACCATTTGTAAGGATTTCCTTTTCCGTGAGCTTATAC

AAAACGACAACAAAATGTAAACATATCAAGGAAAAAGGCATCATCCTTCATTTCCATGCT

GATGTTCACTCTCAGCCGATGCATCTTATCATAAAGACCATGGCTATCCACATGCCTCGT

ATAATCAAAAAAACTTCTACAACTGGACATGTACCTAAGGGACACTTTGTTGTTTATGTT

GGGGAGAAGCAGAAAAAGAGATTTGTTATCCCCCTATCATTCTTGAGCAAACCTCTATTT

CAAGACTTGCTTAGTCAAGCTGAGGATGAATTTGGCTTTAATCATCCACTGGGTGGACTC

ACAATTCCCTGTAGCGAGGATGTGCTTATTGATTTTACTTCTCAATTGAGTAGGATCCGA

GGTGTTCCCTTTCTTAGTTTTGTATAGAACAGGATATTCATCTTGTACAGTAAAGGAACT

AGGAAAGACACATTATTTGTACCATATTACTGAAAATTAGATAGAGGGGCTACTGTCAGC

TGCATCAGTAGATCCCAATTTTGAATGTAATTTTCAGTAGCTGATGTCTAAAAGAAATAC

AACACATTCTTAAGAAAATTAAATCTGTTCAGATTCT

>Gene.88734::Lr_transcript_43000

>LrSAUR18

MAIRMPRIIKKSSTAGDVPKGHFAVYVGEKQKKRFVIPISFLSQPLFQDLLSQAEEEFGFDHPMGGITIPCSEDVFADLTARLNRI

>Gene.97395::Lr_transcript_46053

>LrSAUR19

MAIRMPRIIKKSSTTGDVPKGHFMVYVGEKLKKRFVIPLSFLSEPLFQDLLSQAEEEFDFNYPLGGLTIPCSEDVFIDLTSQLSRI

>Gene.97395::Lr_transcript_46053::g.97395::m.97395 Gene.97395::Lr_transcript_46053::g.97395 ORF type:complete len:87 (+),score=11.50,tr|P33081|AX15A_SOYBN|64.29|3e-32,Auxin_inducible|PF02519.13|1.5e-23 Lr_transcript_46053:4642-4902(+)

GGGTTTGACAACTTCTCATTAGCCTATATAAACACATCCAAAGTCCAAAGAAGGGTTTAC

CATAACAGCTCTCACCAAAACTTGAAACCATACAAAGCTTTTCTCTGCTTTCAGGGTCCT

TACTAATTCTTACCTTCCTTATTTTCTCATCAAGATCATAAAGACCATGGCTATCCACAT

GCCTCGTATAATCAAAAAAAACTTCTACAACTGGACATGTACCTAAGGGCCACTTTGTTG

TTTATGTTGGGGAGAAGCAGAAAAAGAGATTTGTTATCCCCCTATCATTCTTGAGCGAAC

CTCTATTTCAAGACTTGCTTAGTCAAGCTGAGGATGAATTTGGCTTTAATCATCCAATGG

GTGGACTCACAATTCCTGTAGCGAGGATGTGCTTATTGATTTTACTTCTCAATTGAGTAG

GATCCGAGGTGTTCCCTTTCTTAGTTTTGTATAGAACAGGATATTCATCTTGTACAGTAA

AGGAACTAGGAAAGACACATTATTTGTACCATATTACTGAAAATTAGATAGAGGGGCTAC

TGTCAGCTGCATCAGTAGATCCCAATTTTGAATGTAATTTTCAGTAGCTGATGTCTAAAA

GAAATACAACACATTCTTAAGAAAATTAAATCTGTTCAGATTCATTTCTTTAGCCAATTG

GGTTTTCAATATTCTTGTTGGAGAAACTTCTTTACAGGACTCCTTTCATATCTTAAACTC

ATCAACATAAACTTAACACGACAGAAGCTCCAATTCCCGAGTTGTGACATTAATAAATCA

TACCCCATTAGAAATTTTCTTTTTTCTGCTCGTTTAAAACAAATAACTGCCTCTCTGGCT

TGATTTATTAGAATTTCTCTCGACCACCTGAAAAACTTGGATAATTCTGTTCCACGAAGT

TGTTTAATCCATCAGATTATAATGACTTATGATATGAGGGATCAACAATTGTACCTAATT

TTGGTCAACTATTTCTAAATATTTATTAGTACATCTGTATATATTACTGGCAAGTTACCA

AAAATAGTGGTTATGTAATTAACTTATGTTATTAGCACAAAAAGAAAATATAATGTTGCT

TGTTTGCAGGTCATTGATTCGAAACCCGTAACATGTTGTGTTTTACATACTTCCAACGCA

TAAAATCTAAATACCTCGAGTGCGTGTTACCTGGTAGAGTTTTCTTATAGTTGTAGACCA

ACTAGACCAACCAGTTAGGAATCATTTTCTTTTTCTTTATCTTTTCCGTTGAGGAGCTAA

AGTTCACCTTTATCCTCTTTCATCTTTTTCAATTTTTTAAATTTTGCTATACCAGTCTGT

TGTTCAAAGCCTAAGACATAATTTAATTCCCGTAATTAGTCAATTTTGTAAGTCAGTTGG

TTCTGTTAGGAATTCCTAAGATCAGGTGTTTGTTTGTTATAATCTTAGGAACGAGTTCCT

AAACCTTCATAGTATATGCTAATCCACTAGGAACGTTTTGGATTAAGTTCAGTACCGAGC

TAGAAATCACTTGGAGGGCTGGAACATCCCACTTCCCTTTGTTCTACTGATGTCTGTTGA

TGGATATTACTTTTTTGTTAAAAAAAAAATTGCTTTACTAGACAACTATGTTAATGACAA

GAAATATCTATGTGTATGTTGATCACTCAAAGATACATCTCTTCTTTAACAATTTGATGT

GAGGCATGCTATGTTCCAACTTCAGGGAAAAGGTACGGCCTTTATCTTCAACTAATATGT

CCTTGAGTTATCAATATTTGACGATTAGTTGGAAGACAAAGCCCTGTTACTTAAAGTGCT

ATAGTCTACAGATGGTGCCCACGGAATAACTCTTCACTATCAGCAATTAGAATAGGAATG

TCAACTTCTTCTCTGACCATCATATCAAACTCATATGATATCTATATGTGCAAACTTGCC

TTTCCTAACAACAGCTTCTTCAAGATCCTCAGCCAAAAAAAACCTTTGCTATTAAACTCA

ATATTTTCCCCATTAATTACAAACTGGGTTTGATGCTCTTGCTCTCCATCACTTCTCTTC

CATGCATTCAAATGGTGTTCGCATTGGTCGCTACAGGTATATCTCTGTAACATATCTTCA

TCTTGCCTTCAAAACGTCAACTTTGGTTCAAGTGGTTCACGCCCTTTTACATGAATATTC

TGGTTTGCTTTTAAGCTTATATTTGTGCTCTACTTTGGTTGATTTGTTTACCAAGTCATT

GTTTACCAAGAATTCAGCTACGTTGTGCGATGAAATTCCTCTAAAGAACACTGTATGTGA

AAATGCACTATAGTTGTTTAATAGGTTTTTTTTTTTTTTGTCATGCCAGTGTAAAATAAC

AACAACAAACAATGCCTCAATCCCAAGCAAGTTGGGGTCGGTTATAAGAATCCTGACTGA

CCATGCCACTCCATTTAACTTCACCATAGACCCATTATCATGCAAATGTGTGGAAATTAG

TTAGCGAATTCCCCTTTAACGAGAATGACTAGTTAAACTATTTCTATATATTGGGTCTTG

CTGAGTGCCTGAAGTAATTTGACAATGTTTGAATAAGTAATTTAGCTTCTCAAAGGGTGC

TCTAGTCGGACCTTCTAGATGATGTGGTTTATTTTTTTGCTTTCAAAAATGTACGCAAGG

AATAGTATGTGGAATAAAAATGAAAAACCAATGGAACAGAGAAGGGCTTACAGAAAGGCA

GTGAGATAGTTTGGTAGACGTCACAAGATGAGTTAAAGATGCCGATTTTAAAGAAACCAC

ATTCTCCTACTCATGGATTCATGTTGGTCAGCTGAAAATGGTTTGAGGTATTAGTGGTGC

AGCTTGAGGACTTCGGTCGCAAAGATTTTTCAAGCGATTGTGTGATAGCTTTTTCTTCTT

CTCCATCATTCAAGATATTATGTGAATGAATATATTACTGAATCTAATTAGTTAAACTTA

AATTAAAGTAAAAAGAAAAAGTGTTATAAGGTAACTGTTCGCTTTAAAATCATTTTGAGA

AATGTGATAAGGTACTCTTCTTGATGGAGTTGGAATGTTCATAATGAGGTTTGTTACACG

GAAGTGCTGCCACAAGTTTGAATTTCTAGTAAGATTGAGCAAAATGCAAAGATAGTGCTT

CACCATGCTGTAGTTTCTTCGCTAAGAAAATGAGAGCAGTAGCACGCTGAGAACAAATGA

ATTTCTTATCACTGCAAAACCATTTGCAAGGGTACAGTTAACTCAACTCACTGTCTAAAA

GAATTGCTGGGACTAATAGATTACTAACTTTTCTGTTTTCTCCTTTTGAATCAGATTTCT

TTTTCCCTGAGCTTATACAAAACGAAATGTAAAAATATCAACGAAAAGGCATCATCATTC

ATTTCCATGTTGATCTGCATTATCAACCAGCACATCTTTTAAGTTGCTCTTCTATTTTAC

AGTTCTGCCGCCTGATACCATATAATGAACAAACTACCCAATGCTAGGACCATAAATTTT

TAAAGTTTTGATTACTAAACAATTAGAATCATCCAAGAATTGAAATAGTCATGGACTTGC

CTAAATCAATCTCTTAGTTTTTTCTGCATTTTTTGATTTTGCTCTTCATGTAAACTGCTA

AAAGTTGATGGTTTTGTGTGGTTGCTAAATAATCATTTGAAATTCAGAGGCTATTTCTCC

CTGTCCGGAGCTCTTATAGTTTTTCTGTATCTTTTGAGGTCTTTGTCCCTAGTACCATAT

GGGCCATAGTTCTGGATATTCATAATTACTTAGAATATTAAATTTAGTATAGGAATTTAG

ATTCCTAGTAAAGTGCTGTTCGAAGTCAACTTGTTGTTGGATATACATAATCTGATCACT

TAAAGGTGCACTGCGTCAAATTGATTTTCTTGATAAATTTGAGCCAGCATGTTCTTCTTT

TGGATGAGTACCACTCTATATCATAATTGCTTAGCAGATTAAAGATAAGCTTCTTCATTC

TTCTTTTCCTTGTTTCCTACAAAGTTAGTGCATAGGTCCACCCATGCTTAATACAAGTGG

ACACTAATTTGTGTTGTTTTTCTTTGACCACAAACATTGCATTACCTACGACTTCTAATT

GATAAATGAAGCTGGAGAAATATTCTAACCTTATAAATGCTTATTATTTCCAATAATCAT

CACAATTTGACCATTTTATAAGTCTTCAACTGCAGCACCACTTGAAACCATAAATAGTTT

TTTATCGTAAAAACCAACTTGTATCCTGAGCCATTGCTTTGGCATCATGGATAATCAAAT

TTTCGTTCAAAATTCTTAGGATATGCCTTCGTAGATTCGCATTTTGTGATTAATCAGTGG

GACCTTCTGCTTTTAGACTAATCTGATGGTAAAATAATTTGACAATGAGTTGGAAGGCAA

AGCCATGTGATTCTTTAGGTGCTACTGTCTTGAGCTGGTGTCCACAGAGTGCCCTTTTTT

TTGGATAGGTGTGTCCACGTTATGGCTATTCACACATTAGTGGAGTTTGGAAACTTGTCA

TTTGCCTATATAAACACATCCAAAATAGGTTTACCATATCAACTCTCAGCAACACTTGCA

ACCATTCAAAGCTTTCCTCTGCTTTCATATTCCTTACTAATTCTTCCCTTCCTTATTTTC

TCATCTTGATCTTAACGAACAATGGCTATCCGTATGCCTCGTATAATCAAGAAGTCTTCT

ACAACTGGAGATGTTCCAAAGGGCCACTTTATGGTTTATGTTGGGGAGAAGCTGAAAAAG

AGATTTGTTATCCCACTATCATTCTTGAGCGAACCTCTATTTCAAGACTTGCTTAGTCAA

GCTGAGGAAGAATTTGACTTTAATTATCCACTGGGTGGACTCACAATTCCCTGTAGCGAG

GATGTGTTCATTGATCTTACTTCTCAATTGAGTAGGATCTGAGGTGTTTCCTTTCTCGGT

TTTGTATAGAACAGGATATTCAGCTTTTACAGTAGAGGAATTAGGAAAGCCACATTATTT

ATACCATATTACTGAAAATTAGATAGTGGGGCTACTATCAGCTGCATCAGTAGACCCCAA

TTTTGAATGCAATTTTCAGTACCTGATGTCTAAAAGACATGGCACATCACAATTTTGAAT

GTAATTTTCAGTAGCTGATGTCTAAAAGACATACAGCACATTCTTAAGCAAATTAAGTAT

GTTCACATTCATTCTTTAGCCAATTGGGTTTTCAATATTCTTGTTGGAGAAATTTCTTTA

CAAGACTCCATTTGCTCCATAACTTGGGAATTAGAGCTTCCATCATGTTAAAGTCTATGT

TAATTAACTCAAGATATGAAAAGTGACATTAGTAAATCGTACTCCATTAGACATTTTCTT

TTTCTGCTCCTTTTAAAACAAATCTCTACCTCTCTGGCTTGATTTATTAAAATTTCTCTC

GACCATCTGAAAAAACTTGGATAATTCTGATTCAGGAAGTTGTTTAATCCATCTGACTAA

GTTACTTATGTTATGCAACATCAACAATTATACCTAATTTTAGTCTTTATTTCTAAATAT

TTATTAGTATTTACTGGCCTGTTACCAAAAATGGTGGTTATGCCAGTAACTAATGTTATT

AGCAAAATAGAAAAAACTAATGTTGCTTGTTTGCAGGTTATTGAATTGAAACACATAACA

TTTTGTGTTTTACATACTTCAACGCATAAAATCTAAATACTTCAATTGCATGTTGCCTGG

TAGAGTTTTCTTATAATCGTAGACCAACTAGAATAAGAAGTTAGGAATTGTTCTCTTTTT

CTTTATCTTTTTCATTGAGGAGCTAAAGTTCTCCTTTATCCTCTTACATCTTTTTCAATT

TTTTAAATTTTGCCATATCTATCTGTTGTTCAAAGCCTAAGACATAATTTAATTCCTGTA

GTTAGACAAATTTGTAAGTCAATTGGTTCCGTTAAGCATTCCTAAGATCAGGTGTTTGTT

TGTTATAATCTTAGGAACAAGTGTTTGTTTGTTTGTTTTAATCTTAGGAAATAGTTCCTA

AATCTCCATATATATGCAATCCACTAGGAATATTTCATATTAAATTTAGGACCCAGCAAG

AAAGCACTGGGAGAGCTGGAACATCCCACTTTCCTTTGTTCTATTGGCGTCTAGTGATGG

ATATTCCCCCTTTTTTTAAAAAAAATAAAATAATTGCTGTATTACAAAACTATGTTAAAG

ACAAGAAATGTCTAGGTGTATGTTAATCAGGGAAAAGGTCGGGCCTTTATCTTCAACTAA

TTATTCCTAGAGTTATTAATATTGGACGATTTGTTGGAAGACAAAACCGTGTTACTTAAG

GTACTATAGTCTTTTGGGAGCGCCCACGAAATAAGTCTTCACTATCAGCAGGATACTTTG

AGTTGCCATTTGGCCTATATAAACACATTTAGAGAGTGTCTACCACCATAGCTCTCAGCA

ACACTTGAAAGCATTCAAAGAATATTTTTCTTCTTTTGAGGCCTTTTGTATTCTTGTTTT

CCTTCTTTGCTCATATATATTAAATCTAAAAGATCATGGCTATTCGTATGCCTTGTATAA

TCAAGAAATCTTCCACAATTGGAGATTTTCCTAAGGGCCCACTTAGCTGTGTATGTCGAG

GAGAAGCAGAAGAAGAGATATGTGATTTCCTGTATCATTCTTGAGTCAACCTTTATTTCA

AGACTTGCTCTGTCAAGCTGAAGAATAATTCAGTTTCGATCATCCAATGGGTGGTCTCAC

AATTCCTTGCAGTGAGAGGTGTTCATTGATCTCCCAATGCACTCCTGTCAGGCTGCTCCG

AAACTATGATGTGGGTGGTGGCCTTGCACGAATCAAATTTCTCTCGTCTATTGGGCTTTT

TGCATTTGAATTGAGTAAGCCGGTTCATGAACTGGTAATTCAGATGCTCAAAGGGTAATA

CAGTTGGACCTCTAAATAGCGGGGTTTATGTTTCGCTTTCACGATTGAATGTCAGGAACA

GTATGTGGAGTTAGATTGAGAAATCAGGTGAATAGATAAAAAAGGCCTTAAATAAAGGCA

TTGATATAGCCTTGTATATGTCACAAGATGAGTTAAAGATGTCGATCCTAAAGAGCTCCT

ACGCATGGATTCATGTCGCTTAGTAGAAATGGTTTGAGGTTGTGGGGTTCTGAGGACTCT

GGTCGATTAAGCTAAATTGCTAAAAATGTTAAGGGGTTGTTCTTGATGTAGTTGGAATGT

ACTTTATGAGGGTTCAAATATATTGGAATTTGCCAGG

>Gene.110683::Lr_transcript_50652

>LrSAUR20

MSSKMGKSSKIRCIVRISQMLRQWKKRSLISSSKRIAPDVPAGHVAISVGSTCRRFVVRATYLNHPIFRKLLIQAEEEYGFSNHGTLTIPCDELLFEEILRFVSRSGSGRSINIEDFQKSCHARYRNSVENFGDSWPLLGGSTEKSVC

>Gene.110683::Lr_transcript_50652::g.110683::m.110683 Gene.110683::Lr_transcript_50652::g.110683 ORF type:complete len:149 (+),score=-1.09,tr|P33079|A10A5_SOYBN|45.35|1e-16,Auxin_inducible|PF02519.13|5.2e-28 Lr_transcript_50652:74-520(+)

GACACTCTCAATATCATAAAACTCTCTTTTACTTAAGTCATTCTTCTACACAGTTGTCTT

TGCTTGCTTAAAAATGTCGTCTAAGATGGGAAAATCCAGCAAGATCCGTTGCATTGTGAG

GATCAGTCAGATGCTCCGACAATGGAAGAAAAGATCTCTAATTTCTTCATCTAAACGCAT

AGCTCCTGATGTGCCCGCCGGACACGTGGCGATCTCTGTAGGCAGTACTTGCCGGAGATT

CGTGGTGCGAGCGACGTATTTAAACCATCCTATTTTCAGGAAGCTACTGATTCAGGCGGA

GGAAGAATACGGTTTCTCGAACCATGGAACCTTAACTATACCTTGCGACGAGTTGCTGTT

CGAGGAAATCCTTCGTTTCGTATCTCGATCCGGATCAGGCCGTTCGATTAACATTGAGGA

TTTTCAGAAATCATGCCATGCGAGGTACAGGAATAGTGTAGAGAATTTTGGTGATTCTTG

GCCGTTACTTGGTGGATCTACTGAAAAATCCGTGTGTTAAAATACAGGGAAATTTGATTA

TTAATTAGGAGTAGAAACTATTTGTGATCCTGAAAATGGTGATGGCGAAAACAAGGCTTT

TGAGGACTGAGTCGTCCCTAGTTATTTCGTTGACAGACGCCAGATTGTTGTAAAGAGTGT

CTGTTATTCTTTTCTGAAGTTTATATTATATTCTCTCTTTTTTTTTTGCTCAGAAAAAGT

TGTAAAAATACAGTAATTGATACTACAATATACTACTCTTCATGAAATGAAAGTTCCTTA

ATTCTTATTTTTT

>Gene.118121::Lr_transcript_53397

>LrSAUR21

MGSADHKHHHHHLNFHVQVHLPHIHFHHHHHQHGHHGIKELMGIPKGCLPVLVGHDGEELRKFIIPVIYINHPLFTQLLLKGNDLEESELHHDGPINIHCHVEEFRYVEGMIDKETHTTGHHNQHHAAWCYKA

>Gene.118121::Lr_transcript_53397::g.118121::m.118121 Gene.118121::Lr_transcript_53397::g.118121 ORF type:complete len:134 (+),score=15.85,tr|P33079|A10A5_SOYBN|40.00|4e-07,Auxin_inducible|PF02519.13|1.9e-13 Lr_transcript_53397:183-584(+)

GATCCTCAAATCTCAAACCTCAGTCTCAAATTTCATCCTTTTGAACCACTACACACACAA

ATAGATCATTAAAAATGTTCTAACAGATTTAACAAATTCTTTCCCTCATCCTCTTCAAAT

TAACTAGCCATCTCAAACAAATTTCTTGTTTGTTTCATTAGGCTTACCATATTTTTGTAA

CAATGGGTAGTGCAGATCACAAACACCACCATCATCATTTGAACTTCCATGTTCAAGTGC

ATCTGCCTCACATCCATTTTCATCATCACCACCATCAACATGGTCATCATGGGATAAAAG

AACTAATGGGCATCCCAAAAGGGTGTCTTCCAGTATTGGTAGGCCATGATGGAGAGGAGC

TACGCAAGTTCATAATCCCAGTGATATACATCAACCATCCACTCTTCACACAATTGTTAT

TGAAAGGAAATGACCTAGAAGAGAGTGAACTTCATCATGATGGTCCCATCAATATTCATT

GTCATGTCGAAGAGTTTCGCTACGTTGAGGGTATGATTGACAAGGAAACTCATACCACAG

GCCACCACAATCAGCACCACGCGGCCTGGTGCTACAAGGCCTGAGGTTTATGAGGTTAAC

TTCTATACAGTAATAGCGTGAAAGTATTACTTTTATACTACGTACTATTGGATATGAGAT

AAATCCAATGTTTGTTTGTCATCTAGCTATATATATAGCTTGCTAAAAGGTATTTTTTTA

TAGTGATTCGTATGTTCTGTCGGTCTTGTAAATCTGTCTTTACGTTTTCGTGGTTGTATT

GAAATAATCTCTTGTGGTCACTATCTCAATCTCAATAAAGAAAATAGTCCTACT

.

>Gene.120012::Lr_transcript_54049

>LrSAUR22

MMKSTTKMMRKERNNCMLMLRFIMGKLKNHLQLIPKSSRSLEGHVVEFVETPRSNEEVPNDVKEGYFAVFSVNPEEEPKRFIVELHWLTNPSFLKLLKQAEEEYGFEQKGVLEVPCLAADLQKILKLKIGRNITSFAV*

>Gene.126716::Lr_transcript_56446

>LrSAUR23

MAIRMPRIIKKSSTFGDVPKGHLAVYVGEKQNKTYVIPVSFLSQPLFQDLLVKLKKNSVSIIQWVVSQFLAARMCSLISHVA

>Gene.126716::Lr_transcript_56446::g.126716::m.126716 Gene.126716::Lr_transcript_56446::g.126716 ORF type:complete len:83 (+),score=4.02,tr|P33081|AX15A_SOYBN|56.14|1e-13,Auxin_inducible|PF02519.13|1.9e-10 Lr_transcript_56446:114-362(+)

TGACCACAGCTCTCAGCAACACTTGAAAGCATTCAAAGAATATTTTTCTTCTTTTGAGGT

CTTTAGTATTCTTCTTTTCCTTCTTTGCTCATATACATTATATCTAAAAGATCATGGCTA

TTCGTATGCCTCGTATAATCAAGAAATCTTCCACATTTGGAGATGTTCCTAAGGGCCACT

TAGCTGTGTATGTCGGGGAGAAGCAGAATAAGACATATGTGATCCCCGTATCATTCTTGA

GTCAACCTTTATTTCAAGACTTGCTCGTCAAGCTGAAGAAGAATTCGGTTTCGATCATCC

AATGGGTGGTCTCACAATTCCTTGCAGCGAGAATGTGTTCATTGATCTCACATGTCGCTT

GAGGAAGTGAGAAGGAAATAAATCAGTCCCCAGTTTTTGCTTACACAATTTTGTAAAGCT

ATCAAGCATGACAGTTCAGGAGTTAGAAGTAACAGTAATTTGATGTTAGAATGAGGAGAG

TTACCACTCTTTGACTAGGTAGACTAATGAATTTACATCCATAATAAATGGATGTCATTC

CTTTATTCTCATAAGGAAACATTTTTCTACTTCAAAAAAAAAATGAAATAATGAACCTCT

TTGTACTTCTATTGTTCCATATTTATTCCATATATTGATGGAATAATTCATTGCAATGGA

AAATGCTTGCTAAACTTTCTTGGTTTTGTAAGTGCAACTGAGTGCGATAAAAAATATATT

TTG

>Gene.133486::Lr_transcript_58998

>LrSAUR24

MAIRMPRIIKKSSTAGDVLKGHFAVYVGEKQKKRFVVPLSFLSQPLFQDLLSQAEEEFGFDHPMGGVIIPCNEDFFVDLTSRLRK

>Gene.137867::Lr_transcript_60669

>LrSAUR25

MAILRMIKKSSTTRDVPKGHFAVYVGENQKKRFVIPISFLSKPSFQDLLSQAEEEFDFNHPMGGVTIPCSEDLFNDLTSRLRK

>Gene.137867::Lr_transcript_60669::g.137867::m.137867 Gene.137867::Lr_transcript_60669::g.137867 ORF type:complete len:84 (+),score=21.51,tr|P33081|AX15A_SOYBN|66.67|1e-32,Auxin_inducible|PF02519.13|1e-24 Lr_transcript_60669:4183-4434(+)

GGACATTCGAAAGGCACTTGATATGACTATTATCTTGATTTTCAAATGATAGATCGTTTT

GATTATTCTATTGAGTCTCAGATATGACTTAGTTTGTATATGGTTGCTCACTACTCTGCT

CGTGCATGCCTCAATATGTCTTTCACCTAGTCCCGGGCCGGGTATATTTTCGTGCACAGT

TTCACTGCATTGTTCATCGAGTTCCTCACTAGAGGGCCGGGTATGGTATATATATATATA

TATATATATATATATATATATATATATATATATGATATGATGATACGATGAAGTGATTAT

GGCGCCAAGGATGGTACATGATGACTTTATTCACCGAGCCCCATAATGGGCCGGGTATGA

TATATGATATTGACATGCATGATTTATATTTCATAAGGCAAGTGTAATAGTATCTTTGAT

TAACATACTTGTCTCCTGTAATCTCTATTTCAGTGACGATCCTCTTTATTGTATTTCATG

CTTTATATGCTCAGTACATATCTCGTATCGACCCCCCTTTCTTCAGGGGGTTGCGTTTCA

TGCTCGCAGATACAGATAGTCGGTTTGGTGACCCTTCAGTATAGGACTTTTACTCAGCTG

TCTTGTAGAGCTCCGTTGTTCCGGAGTCTAGACTTCTGGTACAGATCTTATGATATGCGT

GCATATATATATATATATATATGCTTATCCAGGGGTACGGCGGGGCCCTATCCCGTCATA

TTTCACTATCGATACTCTTAGAGGTTTGTAGACTTAGGTGTGGGTTGTGTATAAGTTTGT

TCAGCTATGCCTATATATATATATATATATATATATATATATATATATATATATATATAT

ATATATATGCTTATCTAGGGGTACGGCGGGGCCCTATCCCGTCATATTTCGCTATCGATA

CTCTTAGAGGTTTGTAGACTTAGGTGTGGGTTGTGTATAAGTTTGTTCAGCTATGCCTAT

ACGATGTGCTATGGTATTGATATGTATTGGCAGCCTTGTCGGCTTACCTATCATGTTGAT

ATATTGTGGCAGCCTTGTCGGCTTGCGTATCATATTATGTTCTGATAAGTTGTGACTCCC

TAGGAGACCGGTTATTTGGATATATGTATATATGGTGACGTTATGAACCTTCGGAGTTCT

TTTGCAAGTTTTCATATTGATTTTAGCTTCAATTTGACTATATCTAACAGGTACGTATAC

GAGTGTCCAGGTCGGACACTAGTCATGGCCCACGGGGTTGGATCGTGACAATAACTGCTC

AGGTTTAATCATGAGACTTGATAATATGAATATTTTAAATAATGTTGACATGTCAACAGA

AGCTACAAAGATAGCTAAGGTTGAAAATGATTTACAAAATTGGAATATTCCAAGAGAACC

GTTCCGACAAATTTATAAAACAGGAAAATTTGATTTTATAACAAACTATAATATCAAAGC

TTGTGAATCCACAGTTGCTATCAATAGTTCACCACAAACTATTATATTATTATCCATTCA

GGATATAAATAGATGCAAGAAAAATTATAATTTTCTTCACATAGGACTTGTCCAAGTTGC

TGGCAAACCTTTTTACAGATTAGGTTTAGACACACATTTATGTCTCTTACTAAGAGATGA

TAGGTTATTAAACTTTAATGATTCTTTATTAGGAGTTTTACAAAGTAACCTAGCTTATCG

TCGGGTTTACTTTAACTGTTATCCAAACTATTCGGTTGACATCAATGACCAGAATATATT

GGATACATTAACTCTTAATATTAAAACAAGGAACACGAATAGCAAAGTTAATACAAAAGA

AATAGCTGAATATATAGAGTAGTTACAGACTCATGAAAAAAACTTTAGCCCCAAAAGCTA

AAAATATGAGCCCAAAAGGAGTTACTATGCTCATGGAATCAAACCAAGAACATAGTAGTG

GAGTTAAAACTTTAAGCAGAAGAAGAGATATGTGATCCCTGTATCATTCTTAAGTCAAAC

CTTTATTTCAAGACTTGCTCAATCAAGCTAAAGAAGAATTTGGTTTCGATCATCCAATGG

GCGGTCTTACAATTCCCTGCAGAGAAGACGTCTTCATCGACCTTACTTCTCGTTTGAGGA

ACTGAAACAGAAACTAGCTTTCACGATTTTATAAGAGGAGCAAAGCACGATCTGTACAGT

TGAGGGGTTAGAGAAAACTGTAGTTGATGTTAGATTGAGGGACATTCAGACTAGGCAGAT

TAGTGAACTTATATTAACAAACCCTGATTGCTATTCATGTCAAAGAGATGAATGACTCTT

TACTTTTTTTGTACTTCAAATATATTGAAAGTATTGAATCTTTTTCCACTTGATGGTTCC

ATTCAGTTAGCTTTGGTGTTTTTTTCTCCTTATGATTCCTTACTGTCGAGTCCCGTGGTT

TGCTAACAATTATGAGGCAGAATCAAGGGATGTCTAAAGTGGCCCAAACTTGACGATAAT

CAGTCACTTTGTTGTGTTACTACTAAAATAGGAAACTGTGAAGGCGAAAGAGCTTTGCTT

AATTTCCTAAGACATTGCAAAGCTACTAGTTAACAACGTGATCGAATGCATTTAAGTTAG

TCTAAACATCTAATTTTTATTGATGATATAGTCATGAAAGTAGGACCATATCTATTATTC

ACCAAAATGACCTCAAATTATAAGTACTGCACAAAAGAAAATAGATGGTAAAGCCATATG

ATCCTTTATGTGCTAGTGTCTTTCATGTTATCAACCTGTATTCGAAAAATTATTTTAACC

TGAAACTGAAAACTAAAAAAAGCAAATTCTGAAATGTAGAAGAACAGTATGTTAAAAATA

TAAGGAATTAATTGAGCCCACTGAATGCACTGTGTGTCCTTAAGGAAATTATTCCCCTCA

ATGTACCCGGTGTTGTGGAATATATCCTCCCAGGATAGACCGAATTACTCAGCGTTGTAG

CGGTACAACAAACTCAGGTGACGGCGAATCACTCGACAACAGTACATCACACGAGAATTT

ATTTTAGTGCAAGAAAGAAGAGAGAAGTTCATAATTTTCGTAAAGAAAATTGGATGGACT

GGCCAGATTTATATAGCAATTGGGTAGAGTTAAAGTGAAAAGATGCAACTCAAAAGTTGC

AACTTTTCAGTTAAGTTGTATCTTTTCAGAAAAATTTCCCATTCAGCTTAACTTAAACCC

AACATTTCACAGACATCATTTAAGTAATTTTTACCACTTATTATTACCATGATCAGGTAT

CATTCTCTCTCTTCACATATATGATCTGAAATCAAATGTTTGTAATCACCACACTGATTA

GGTAGCCTATGCGTGGCCTTTCTGCTATGTACATTCAATTTAATGACAAGTAGAACGATA

AGTGACAGTATGACGATGGCATGGCAAAGCAGCAATATTACAATCTTTGTTGCACTTCCC

AAACCTCCACATATGGAGATCTCATATATTTTGGATCAAGTTATAGTCTGAAGCTAATAA

GCACTTGGAACTGGAACTTTATGGTCATATGAGTTTTTTTATTTTCTCAGGGATAACGCT

TCCAACAAAAGATGTGTACCTAGACATAAATAATAAAATGGTATTGCTCTTCTTTAGCAA

TTTGATTGTGAGGGATATTTTGCTCTAAGGTCAAAGAAAAGGTAGGGCCTGCTGTCCTTG

AACTAAGGCTTTTTAAATCTGAACAAAGCTAGGAAGCACTTGGCTTCTCTCACGTTACTT

GTCGTTTTGGGATAGTAATTGTTAACATTTTATGTATCCCAATATGGTGCTTACTATAGA

TCCATTTTAATGGCTATATATGTCCAGTTGTATGTTTTTATCCCAGTGGGCCAGGCATAA

ATAAGCTCCAGGTTATCTCTTCTAAAAAATTATACAATGATTAATGCTTTGTCCAGAGTT

TCAAAATGAAGTAGGGCTTATTGTTCTTGAACTAATAGTGCAAAGTATTAATATTTGGAA

GGCAAAGCATGTGATACTTCAGTGCCGTTGTCTTCTGCTGGTGCCCACAAATTCCTATGC

AACACATATCAGCAGTGCTGAATGAGTTGCCATTGCCTATATAAACATATTCAAAGACGT

ATCTACCACCACAGCTCTCAACAAGCTTCAAAGTATTCAAAGCTTCCTCTGCTTTGAGTT

CTTTAGTAATTCTTTGCTTTTCTCAACTGTAACTAAAAGATCATGGCTATTCTTCGTATG

ATCAAGAAGTCTTCCACAACTAGAGATGTTCCCAAGGGTCACTTTGCTGTGTATGTTGGG

GAGAATCAGAAGAAGAGATTTGTGATTCCAATATCATTCTTGAGCAAACCTTCATTTCAA

GATTTGCTTAGTCAAGCTGAGGAAGAATTTGACTTCAACCATCCAATGGGTGGTGTGACT

ATTCCCTGTAGTGAGGATTTGTTCAATGATCTCACATCTCGCTTGAGGAAGTGAGAAGGA

AACCAATCCCCATTTTTTGCTTACACAATTTTGTAGAGTTGTAAATCAGGATCTCCAGTG

TAAAGTTCAGAGTTAGAAGAAACACTAGGTAGACTTGGGAAACATTTTTTACTACTTCAA

TAGAAATTGAAGTAATGAACCATTTTACTTCTATCGTTCCGTTGTATTCCATATATTGAT

GTAATCAATTCATTTCAGTAG

>Gene.142508::Lr_transcript_62376

>LrSAUR26

MKVKKGWLAIQVGLEEEDGGIQRFVIPISYLYHPLLQKLLDKAHDVYGYHVDGPLKLPCSVDDFLHLRWRIEKEPNRSHHHHHHKNIHQHLPSTLSFHSC

>Gene.146242::Lr_transcript_63699

>LrSAUR27

MAIHMPRIIKKTSTTGHVPKGHFVVYVGEKQKKRFVIPLSFLSEPLFQDLLSQAEDEFGFNHPMGGLTIPCSEDVLIDFTSQLSRIRGVPFLSFV

>Gene.172674::Lr_transcript_73228

>LrSAUR28

MEFDKLCGKSKKGLITKTWKRCTSFGSFGRKNNQQSLSIKSKPWTEGLSTGTGKKNRVVPEGCFSVYVGHQRQRFVIRTKYLNHPLFRMLLEEAESEFGYSSEGPLVLPCDVDIFEKLLMEMDDSDEVDHRRGCSFAAKTHDSYYRLLSPTASAFNKFSF

>Gene.174128::Lr_transcript_73754

>LrSAUR29

MKVKKGWLAIQVGLEEEDGGIQRFVIPISYLYHPLLQKLLGKAHDVYGYHVDGPLKLPCSVDDFLHIRWRIEKEPNRSHHHHHHKNIHQHLPSTLSFHSC

>Gene.174128::Lr_transcript_73754::g.174128::m.174128 Gene.174128::Lr_transcript_73754::g.174128 ORF type:complete len:101 (+),score=14.62,tr|P33083|AX6B_SOYBN|47.06|6e-13,Auxin_inducible|PF02519.13|2.1e-15 Lr_transcript_73754:132-434(+)

GACGACGCACCAATATTATCTCCTACTCACATCCCTCACTACTCGCTCTACATTAACTTC

TTCAATTGGTTAATAGTCCATTTGAAAAAAAAAATAAGGAATAACACTATTAGTGGTTTA

AGATCGAGAAAATGAAGGTGAAAAAGGGATGGCTAGCAATTCAAGTAGGATTAGAAGAAG

AAGATGGTGGGATTCAAAGATTTGTTATTCCAATTTCATATCTTTATCATCCTCTTCTTC

AAAAGCTTCTTGGTAAAGCTCATGATGTTTATGGCTACCATGTTGATGGACCCTTAAAGC

TACCATGCTCCGTCGACGATTTCCTCCATATACGGTGGCGGATTGAGAAGGAACCTAATC

GGAGCCACCACCATCACCACCACAAAAACATCCACCAACACCTTCCTAGCACTTTGTCCT

TTCACTCTTGTTGATGATGGATGAAGAGCTTCTTTATTTGTAAAGGTGTGGCTTTTTTTT

TTTCCTTTTCTTGTTTTATTTTTCCTTCTTCTAAAGAAGTGGTGTTTTGACAAAGCAAAA

CTTCAAAGCATTACCGTGGCATGGTGATTTAGGGTTTGAGTCTTGAGAATGGACTCATCT

TTGATAGGAAGCATTTTACGTTCAAAATAGGATTTTCTTGCGCGCGAATCTGAGCTAATC

ATAGCTCAAGGCAAATACTGAATGCCAGATGAAAATCAAAAGGAGCCGCATAAATGTTGA

TTTATTTTTATGGTGTGAGACATTTTGGGAAAAGCCACGTGACCAGATTTGGTTCAAAGC

CATGTTAAGAATATTTTTGGGTGGCTTAACACAACAAAATGATATTAGAGTCAAATGTTT

GATGAGACGAACATTAGAGTTGTGGCAAAGTGATCAACATGGAATGACCTTCTTAGTGTC

TTTATCCGTATATGGACTGAATAATTATCTTTACTCATAGTTTTAAAATGCATACGCAGA

AAGGAATCTGATGATGTGGATGACATACACTTATGTTGAATCATATGGAACATAATTCGA

GCACGAAATTACGTCTTTTTTTTTTTAAACTATTGATAGGGAGATAGAGTAGAGATTTGA

ACTTTCTATTATTGAGATCTATCCCTGTGAGCATCTTCTTTTTACTTTGTGTGCTCGATG

GAGATGATGACGAATCATGTTGCTCATAATGAAAAAAGGTTC

>Gene.193632::Lr_transcript_80939

>LrSAUR30

MGGGERSLLHLPHLHIHQGKKKTSDVPKGYLAIKVGQEEEEQQRFVVPVSYFNHPLFIQLLKEAEEVYGFHHKGTITIPCHVEQFRSIQGKIDKHHHHNHHHHIHVPCFRA

ne.193632::Lr_transcript_80939::g.193632::m.193632 Gene.193632::Lr_transcript_80939::g.193632 ORF type:complete len:112 (+),score=20.61,tr|P33083|AX6B_SOYBN|47.44|6e-17,Auxin_inducible|PF02519.13|2.5e-22 Lr_transcript_80939:252-587(+)

GACCTAGCTATTCCATATTATTATGCTTTTTTCTCCATATTCCTATATGTTACAAATTCA

GCCATGACAAACTTCCCAAGGTCTCTCAGATATTCTTCTTTAGCCCTTTTCAAACAGGCT

ATCTCTGCTAATCCTTGGCCTAATTCTTCCCTTCTTCTCTGAAAAAAAAAAAAAACTTTT

TTCTTCTCTGTATAATTTTGCTAATTCCTAGTACTAGTATTTGTTTTTAATTTGCAGGAT

TAATTAAAGCAATGGGTGGTGGAGAAAGGAGTCTTTTGCACTTGCCACACCTCCATATTC

ATCAAGGGAAGAAAAAGACCAGTGATGTGCCAAAAGGGTATCTGGCGATAAAAGTAGGGC

AAGAAGAGGAAGAACAACAGAGATTTGTTGTTCCTGTTTCTTATTTCAATCATCCGCTTT

TTATTCAGTTACTCAAGGAAGCTGAGGAAGTTTATGGTTTTCATCACAAGGGAACTATTA

CTATTCCTTGTCATGTTGAACAGTTTCGAAGTATTCAGGGAAAGATTGATAAACACCACC

ACCACAATCACCACCATCATATTCATGTCCCATGTTTTAGGGCATGAGATTCTAATTTTT

GTTCGGTGGAGGCTGTTAATGATGATGACGACAACTTTTTATGTTCCCAATGGTTTGTTA

TTGGTGTCTGAATTTGTTAGCTAAACTGCTTACTAGTGAAGTGGAATTAAAGTTACTTTT

TCCTGTTCTTTTCGTATTCTCTCCTCTTTTGGTTATTAATACCTTGTTTGGATGGCTGTT

ACTTAGTGTATTGCCTGCTATTAGTTTAAATACAATATTTATAATTTGATTGTTACTTAA

AATTTTATTGTATTGTATTGTATCATTAAATTTATT

>Gene.206924::Lr_transcript_85748

>LrSAUR31

MRKFRGFVLKHRVTTLFRCIFRRRRWATARYHRLDQLPSWNGPTKSFSRFLNWTQRVKTRAKAICSKAHCYGSGLGYMHVGQDPVEDESVTVPKGHLAVYVGQKDGDYKRVLVPVIYINHPLFSELLREAEEEYGFNHPGGITIPCRISEFEHVQTRIKQGRVG

>Gene.206924::Lr_transcript_85748::g.206924::m.206924 Gene.206924::Lr_transcript_85748::g.206924 ORF type:complete len:165 (+),score=0.33,tr|P33080|AX10A_SOYBN|44.32|1e-19,Auxin_inducible|PF02519.13|5.6e-24 Lr_transcript_85748:358-852(+)

GGATCTCTAATTCACATACTCCACATCTTCCATTTCACTTCTCCCCTCCACCCAAAGGCA

AAAAAAAAAAAAAAAAAAAAAAAAAAAAAGAACTTGCAAAATTGCTCTTTTATCACCAAT

TTTATCCTGCAATATCTTTTCTTCGTTTTCTGATGAAAGACTCAACTTGTATTAGCCTTG

TTCAATACACCAATTTCTTTTTCTGTAATTTGCCTTACACTTTTCAGGTTAACTATGTCC

ATAACTGATCCCTGACCATTTTCTTAAAAACCCCACACACAAAAACAAAAAGCTCTTTTC

CTTGTAAAAATATTTCATCTTATCTTCTTTCAGTTTTTCCTAGTACAAATAGTAAACATG

CGTAAATTCCGAGGTTTCGTGCTCAAACACCGTGTCACTACACTTTTCCGGTGCATTTTC

CGGCGAAGACGGTGGGCAACGGCGAGGTATCACCGGCTGGACCAACTTCCAAGTTGGAAT

GGACCCACAAAATCATTTTCTAGGTTCTTGAACTGGACCCAACGTGTCAAAACAAGAGCC

AAGGCTATTTGCAGCAAGGCTCATTGTTACGGGTCGGGTCTGGGTTACATGCATGTCGGG

CAAGACCCAGTTGAGGATGAAAGTGTAACGGTGCCAAAAGGTCATTTGGCTGTTTATGTG

GGACAGAAAGATGGTGATTATAAAAGGGTTTTAGTTCCTGTTATTTATATTAATCACCCT

TTGTTTAGTGAGTTGTTAAGGGAGGCTGAAGAAGAATACGGGTTTAATCACCCGGGTGGT

ATTACGATACCTTGTCGGATCTCGGAGTTTGAGCATGTTCAGACCCGGATTAAGCAGGGT

CGGGTCGGGTGACCCGGAAAGTCTGAACATGGAAGTGATGCATGCAATATGCTACCATTT

GGATTGTTTATGATGATGATGACGATAATTGTTAAGAGACAGTTGAGAGTTGTTTATCAG

GGATTTTTGTAGTTTTTTTTTTTTTTTTTTTTTTTTTTTTGGGTTTTGAAATTTCGGTGG

TTTCATTTCTGATGTAAGAAGAGTAGTTTATTGATGTACAGTATTAGATGAAACATCTTT

CCAGTC

>Gene.228330::Lr_transcript_93488

>LrSAUR32

MAILRLIKKSSTTRDVPKGHFAVYIGETQKKRFVIPISFLSEPLFQDLLSQAEEEFGFDHPMGSVTIPCSEDLFIDLTSRLRN

>Gene.228330::Lr_transcript_93488::g.228330::m.228330 Gene.228330::Lr_transcript_93488::g.228330 ORF type:complete len:84 (+),score=17.20,tr|P33081|AX15A_SOYBN|65.79|7e-32,Auxin_inducible|PF02519.13|1.4e-24 Lr_transcript_93488:101-352(+)

GACAGCTCTCAGCAAACTTCAAAGTATTCAAAGCTTTCTCTGCTTTTGAGTTCCTTAATA

ATTCTTTTTCTTTTCTCATCTCTACTAAAACTAAAAGATCATGGCTATTCTTCGTCTAAT

CAAGAAGTCCTCCACAACTAGAGATGTTCCCAAGGGTCATTTTGCTGTGTATATTGGGGA

GACACAGAAAAAGAGATTTGTGATCCCCATATCATTCTTAAGTGAACCTTTATTTCAAGA

CTTGCTTAGTCAAGCTGAGGAAGAATTTGGCTTTGACCATCCAATGGGCAGTGTCACAAT

TCCTTGCAGTGAGGATTTGTTTATTGATCTCACATCTCGCTTGAGGAATTGAGAAGGAAA

CCAGTCACCACTTTTTGCTTACACAATGTTTTATTGTTGTAAGCCAGGATCTCTACTGTG

TAGAGTTCAGAGTTAGGAGAAACACTAGGTAGACTAAGGACACATTTTTCTACTTCAAAA

GATTTTGAAGTAATGAAATTTCTTACTTGTATGGTTCCAATCAATTCATTGCAGTAGAAT

TTTTTTTCC

>Gene.236195::Lr_transcript_96479

>LrSAUR33

MAILRMIKKSSTTRDVPKGHFVVYVGETQKKRFVIPISFLSEPLFQKLLSQVEEEFGFDHPMGGVTIPCSEDFFIDLTSRLRK
