# Supplementary material for: Identification and expression analysis of the small auxin-up RNA (SAUR) gene family in Lycium ruthenicum
Source: PeerJ. 2023 Sep 7;11:e15941. doi: 10.7717/peerj.15941 (PMC10493089; doi:10.7717/peerj.15941)
Supplement: Table S1 [file peerj-11-15941-s002.docx]

Supplementary table 1 primers of Real-time qPCR

| Primer | Sequence(5’-3’) |
| --- | --- |
| S2F | ATTTGAAGGGATGGGCCGTT |
| S2R | ACCAATCGCCAATGCTCAGA |
| S4F | AAATCACAAGCGATGGCAGC |
| S4R | GGGCTCTTTAAGGACCGCTT |
| S5F | ACATCCAAAAAGCTTCCTGCT |
| S5R | TAGTGGCACCAGTCTCCTCA |
| S6F | GGAAGGTTGAGGACACGTCA |
| S6R | GGTCTTGGGGTGATATCCTGG |
| S7F | GGAAGGTTGAGGACACGTCA |
| S7R | GGTCTTGGGGTGATATCCTGG |
| S8F | ACCAGTTGTGGCGGAGTATG |
| S8R | CCAACTTTAGTGAGATGCGGC |
| S9F | GAGGACACGTCAGTTCCTGT |
| S9R | TAGGGTGGTGTCCCGGTAAT |
| S10F | GAAGCGGAAAAGCCAACTCA |
| S10R | CTGGAACTGACGTGTCCTCAA |
| S11F | ACTTGCCATATCGGCTTGTGA |
| S11R | CTCAGATGATGGTTCTGACCCC |
| S12F | TCTTGTTGGCAACTCAAGAAGG |
| S12R | GTTGTGGGGGTCAGCATTTTC |
| S13F | TTCCGAAAGGCCACTTTGCT |
| S13R | ACATGGAATTGTGACACCGC |
| S17F | ATGGCTATCCACATGCCTCG |
| S17R | AAGCACATCCTCGCTACAGG |
| S19F | TGGTGCCCACGGAATAACTC |
| S19R | GTAGCGACCAATGCGAACAC |
| S20F | CACGTGGCGATCTCTGTAGG |
| S20R | GAACAGCAACTCGTCGCAAG |
| S21F | GGAGAGGAGCTACGCAAGTT |
| S21R | GGTGGCCTGTGGTATGAGTT |
| S23F | CCACAGCTCTCAGCAACACT |
| S23R | TGCTTCTCCCCGACATACAC |
| S25F | ATACGAGTGTCCAGGTCGGA |
| S25R | TGTCGGAACGGTTCTCTTGG |
| S29F | TACGGTGGCGGATTGAGAAG |
| S29R | CACCATGCCACGGTAATGCT |
| S30F | CAGCCATGACAAACTTCCCAAG |
| S30R | TCTCCACCACCCATTGCTTT |
| S31F | TTCCGAGGTTTCGTGCTCAA |
| S31R | GAGCCTTGCTGCAAATAGCC |
| S32F | TGTTCCCAAGGGTCATTTTGC |
| S32R | GACACTGCCCATTGGATGGT |
| LrEF1a-F | CCATACCAGCATCACCATTCTTC |
| LrEF1a-R | GTCACACTTCCCACATTGCC |
